# SARS-CoV-2 PCR positivity rate and seroprevalence of related antibodies among a sample of patients in Cairo: Pre-wave 2 results of a screening program in a university hospital

**DOI:** 10.1371/journal.pone.0254581

**Published:** 2021-07-15

**Authors:** Samia A. Girgis, Hala M. Hafez, Hoda Ezz Elarab, Basma Sherif, Moshira H. Sabry, Iman Afifi, Fatma Elzahraa Hassan, Amira Reda, Shaimaa Elsayed, Asmaa Mahmoud, Petra Habeb, Ihab S. Habil, Rasha S. Hussein, Isis M. Mossad, Ossama Mansour, Ashraf Omar, Ayman M. Saleh, Mahmoud El-Meteini

**Affiliations:** 1 Professor of Clinical Pathology, Faculty of Medicine Director of Infection Control Unit and Vice Director of Ain Shams University Hospitals, Cairo, Egypt; 2 Professor of Clinical Pathology, Faculty of Medicine, Head of Clinical Microbiology Unit, Ain Shams University Hospitals, Cairo, Egypt; 3 Department of Clinical Pathology, Ain Shams University Hospitals, Cairo, Egypt; 4 Infection Control Unit, Ain Shams University Hospitals, Cairo, Egypt; 5 Department of Community, Environmental and Occupational Medicine, Faculty of Medicine, Ain Shams University, Cairo, Egypt; 6 Vice Dean of Faculty of Medicine, Ain Shams University, Cairo, Egypt; 7 Dean of Faculty of Medicine and chairman of board of Ain Shams University Hospitals, Ain Shams University, Cairo, Egypt; 8 Vice President of Ain Shams University, Cairo, Egypt; 9 President of Ain-Shams University, Cairo, Egypt; University of Cincinnati College of Medicine, UNITED STATES

## Abstract

**Background:**

Research has revealed that asymptomatic and pre-symptomatic infections are important contributors to the transmission of SARS-CoV-2 in populations. In Egypt, the true prevalence of infections is veiled due to the low number of screening tests. The aim of this study was to determine the SARS-CoV-2 PCR positivity rate as well the seroprevalence of the SARS-CoV-2 antibodies before the ultimate development of a second wave of the epidemic in Cairo, Egypt.

**Methods:**

Our study was carried out between May 5 and the end of October 2020. It included all patients requiring admission to Ain Shams University hospitals. An interview questionnaire was used to collect demographic and clinical data. Laboratory tests for all participants included RT-PCR and total antibody assay for SARS-CoV-2.

**Results:**

A total of 4,313 subjects were enrolled in our study, with females representing 56% of the sample. Adults and middle-aged individuals represented around 60% of the study sample. The positivity rate of SARS-CoV-2 PCR was 3.84% (95% CI 3.29–4.48), and the SARS-CoV-2 antibody seroprevalence was 29.82% (95% CI: 28.16–31.51). Males showed a higher risk for getting the COVID-19 infection, while middle-age group had significantly higher antibody seroprevalence rates.

**Conclusion:**

SARS-CoV-2 infection imposes a high burden on the community as detected by high seroprevalence rates.

## Introduction

The new coronavirus, officially named “severe acute respiratory syndrome coronavirus 2 (SARS-CoV-2),” infected around 155 million people worldwide by the beginning of May 2021, with more than 3 million related deaths [1], and the numbers are steeply increasing every day. In Egypt, the first peak of reported cases was recorded by June 2020, with about 65,000 infected cases and 2,789 deaths. Reported cases were stable over the summer season, with around 6,000 cases per month. The infected cases began to surge by November 2020 and reached around 231,803 by the beginning of May 2021, with 13,591 deaths [2]. Egypt is ranked 68th in the world in terms of COVID-19 infections and 8th in terms of COVID-19 deaths. The capital, Cairo, with its high-density population, took the lead among Egyptian governorates in terms of the number of COVID-19 cases, according to data declared by the Egyptian Ministry of Health [3].

COVID-19 surveillance in Egypt mainly depends on the reported PCR detection that is usually carried out in symptomatic cases. According to the Ministry of Health’s announcement in October 2020, the number of daily PCR tests can hardly reach 6,000 when compared to other Arabic countries such as Saudi Arabia and United Arabic Emirates, during the same period [4]. Studies show that about 30%–60% of COVID-19 patients have mild or no symptoms and still can spread the infection [5]. Previous studies have suggested that only a small fraction of asymptomatic persons may eventually develop symptoms [6–8]. These facts add to the struggle of estimating the magnitude of COVID-19 in a community.

Many researchers are attempting to estimate the rates of infection in the community through epidemiological models [9, 10] or structural assumptions [11]. With limited testing availability and a high proportion of mild and asymptomatic infections, there is an under-ascertainment of SARS-CoV-2 infections through passive case reporting [6–8]. In such cases, seroprevalence surveys of SARS-CoV-2 antibodies are important for refining estimates of infection and transmission [12]. Moreover, seroprevalence studies can provide information on risk factors for the disease, such as a patient’s age, location, or underlying health conditions. Furthermore, they could show significant medical data on immune reactions to the virus and provide an understanding of the immune response following infection of SARS-CoV-2 [13].

In hospital settings, the SARS-CoV-2 infection presents a great challenge, where it is highly infectious during the pre-symptomatic period in patients. The nosocomial transmission of COVID-19 to healthcare workers and other patients can have serious impacts on hospital operation, including the suspension of new admissions and the closing of hospital wards. Preadmission screening by PCR is a policy recommended by different agencies, including the Centers for Disease Control and Prevention (CDC) in the US and the Public Health England guidelines, depending on testing capacity and disease prevalence [14].

By the beginning of May 2020, the Ain Shams University (ASU) hospitals in Cairo adopted a universal screening program for all patients requiring admission to the hospitals. The screening process included PCR testing and total antibody assay prior to admission.

With scarce data available on the epidemiology of COVID-19 in Egypt, the aim of the present research was to determine the SARS-CoV-2 PCR positivity rate as well the seroprevalence of SARS-CoV-2 antibodies before the ultimate development of a potential second wave of the epidemic in Cairo, Egypt. The findings are based on the results of a universal screening program for patients in ASU hospitals in Cairo.

## Subjects and methods

The current study was carried out between May 5 and the end of October 2020. The protocol of the research was approved by Ain Shams University Faculty of Medicine Research Ethics Committee [FWA00017585], research approval number (FMASU R/2020). Positive cases were reported to the Ministry of Health and Population (MOHP). The guidelines of isolation and treatment protocol of the MOHP were followed. A written informed consent in Arabic was obtained from participants or their guardians in the case of minors (Arabic and English translations of the consent form are attached in the supplementary materials).

### Study setting

This study took place in ASU hospitals. It included seven hospitals (surgery hospital, internal medicine hospital, pediatrics hospital, obstetrics and gynecology hospital, emergency hospital, and geriatrics hospital). The hospitals have a capacity of more than 3,000 inpatient beds and serve about 1.5 million patients annually.

### Study population

All patients needing admission to ASU hospitals were eligible for the study.

### The hospital screening program

By the beginning of the epidemic in Egypt, ASU hospitals established a symptom-based screening clinic for all patients seeking hospital services. SARS-CoV-2 PCR and total antibody assay were done for all patients requiring hospitalization.

### Study methods

Every enrolled patient was subjected to:

1. An interview questionnaire. The study questionnaire included:
  - Background characteristics (age, gender, residence, and contact details)
  - History of contact with a COVID-19 case
  - Clinical data: Temperature was measured on admission as part of the preadmission screening. Patients were asked about other symptoms, e.g., cough and sore throat.
  - History of comorbidities: Self-reported diabetes and hypertension
2. Laboratory Tests: Reverse Transcription Polymerase Chain Reaction (RT-PCR) and total antibody assay for SARS-CoV-2. Tests were done only once before admission. PCR was repeated for initially negative tests only if the patient developed suspected symptoms.

### Specimen collection and handling

1 Following the recommendations of the US CDC, combined oropharyngeal and nasopharyngeal swabs were collected from studied patients using sterile swabs with synthetic tips (dacron/nylon) and plastic, flexible shafts.

The swabs were rubbed against the posterior pharyngeal wall and tonsillar pillars, and then, the same swab was inserted into the patient’s nostril while tilting the patient’s head 70 degrees, and it passed slowly parallel to the palate until resistance was encountered. The swab was left in place for a few seconds to allow for secretion absorption and then was slowly removed while being twisted. Finally, the swab was immersed into a sterile tube containing 2 mL of viral transport media and was immediately transported to the laboratory at a temperature of 4±1°C.

2 Serum samples: A sample of 3 ml whole blood was collected from each patient by peripheral venipuncture on a clot activator and gel separator vacutainer tube. The tubes were immediately centrifuged, and the separated serum was used to measure the SARS-COV2 total antibodies using the Elecsys® Anti-SARS-CoV-2 immunoassay (ROCHE).

### I-Detection of SARS-COV2 RNA by reverse transcription real time polymerase chain reaction (rRT-PCR)

#### Nucleic acid extraction

Samples were mixed well by gentle vortex and a volume of 300ul was used to extract the viral RNA. The viral RNA was automatically extracted by binding to the surface of magnetic beads using the Chemagic 360 automatic extractor (Perkin Elmer, Germany) and the Viasure RNA/DNA extraction kit (*CerTest Biotec*, Spain). Usually, RNA was processed immediately after extraction, except in cases where the PCR equipment was busy, and we had to wait less than 2 hours to process the samples; therefore, we stored the extracted RNA at −20°C for less than 2 hours in exceptional situations, as per manufacturer recommendations.

#### Detection of SARS-CoV2 RNA by rRT-PCR

SARS-CoV-2 Real Time PCR Detection Kit (*CerTest* Biotec, Spain) was used for the detection of SARS-CoV-2 in the respiratory samples. Detection is done in one step real time (RT) format, where the reverse transcription and the subsequent amplification of the specific target sequence take place in the same reaction well. The isolated RNA target is transcribed, generating complementary DNA by reverse transcriptase, which is followed by the amplification of a conserved region of ORF1ab and N genes for SARS-CoV-2 using specific primers and a fluorescent-labeled probe.

#### The amplification protocol

The thermal cycler was programmed as follows:

**Table:** 

**Cycles**	**Step**	**Time**	**Temperature**
1	Reverse Transcription	15 min	45°C
1	Initial denaturation	2 min	95°C
45	Denaturation	10 sec	95°C
	Annealing/Extension (Data collection)	50 sec	60°C

#### Quality control

Samples were processed only once. In each run, a positive and a negative control were included to allow for correct interpretation of the results. Moreover, the presence of an internal positive control (IPC) in each run ensures the correct performance of the amplification mix.

#### Interpretation of the test results

The sample was considered positive for SARS-COV2 when the obtained Ct value was less than 38 and the IPC showed an amplification signal. On the other hand, a negative sample would have no amplification signal, but the IPC would be positive to exclude the inhibition of the PCR reaction.

The absence of a signal in the positive control or the presence of amplification in the negative control would denote invalid test results.

### II-Detection of SARS-COV2 total antibodies

The Elecsys® Anti-SARS-CoV-2 is an immunoassay for the invitro qualitative detection of antibodies (including IgG) to SARS-CoV-2 in human serum and plasma. The assay uses a recombinant protein representing the nucleocapsid (N) antigen in a double-antigen sandwich assay format, which favors detection of high affinity antibodies against SARS-CoV-2. The test is intended as an aid in the determination of the immune reaction to SARS-CoV-2.

A volume of 20 μL of the patient serum was incubated with a mix of biotinylated and ruthenylated nucleocapsid (N) antigen. Double-antigen sandwich immune complexes are formed in the presence of corresponding antibodies. After the addition of streptavidin-coated microparticles, the double-antigen complexes bind to the solid phase via the interaction of biotin and streptavidin. The reagent mixture is transferred to the measuring cell, where the microparticles are magnetically captured on the surface of the electrode. Unbound substances are subsequently removed. Electrochemiluminescence is then induced by applying a voltage and is measured using a photomultiplier. The signal yield increases with the antibody titer.

A cutoff index of <1.0 is considered non-reactive, whereas a cutoff index of ≥1.0 is considered reactive.

### Statistical analysis

Data were validated, cleaned, and entered into a spreadsheet. Qualitative data were presented in frequency and related percentages. The level of antibodies was presented by median and interquartile range with the Mann–Whitney U test used for comparison. Unadjusted frequency of positive screening among the total was calculated with a 95% confidence interval. Given that SARS-CoV-2 PCR sensitivity was reported to be between 71%–95% [15], the PCR positivity was adjusted for test sensitivity for both scenarios with a specificity of 99.9%. The antibody seroprevalence was adjusted for kit sensitivity and specificity. According to the manufacturer’s package insert, Elecsys®.Anti-SARS-CoV-2 exhibits a high overall clinical specificity of 99.81% with no cross-reactivity to the common cold coronaviruses and a sensitivity of 100%. We used the Clopper–Pearson exact method to calculate 95% confidence intervals.

Comparison between groups was done using a Chi-square test with a P value of 0.05 as the level of significance. The odds ratio was calculated for the estimation of risk with a 95% confidence interval. Logistic regression was used for adjustment of the confounding factors.

SPSS program version 15 was used for the analysis. Epitools Epidemiological Calculators. Ausvet. was used for adjustment for tests’ sensitivity and specificity. Available at: http://epitools.ausvet.com.au

## Results

The current research enrolled 4,313 subjects during the study period. A total of 4,008 and 2,951 patients had the PCR test and the antibody assay, respectively. Females constituted 56% of the study sample. Adults and middle-aged individuals represented around 60% of the sample. Most patients (91.3%) did not complain about any related COVID-19 symptoms (Table 1).

**Table 1:** Characteristics of the study group.

	No. (%)
**Total no**	4313
**Age (years)**	
** <18**	928 (21.5)
** 18–**	1356 (31.4)
** 40–**	1214 (28.1)
** ≥60**	815 (18.9)
**Gender**	
** Male**	1885 (43.7)
** Females**	2428 (56.3)
**Hospital**	
** 1. Obstetrics and gynecology**	703 (16.3)
** 2. Oncology**	49 (1.1)
** 3. Surgery**	1463 (33.9)
** 4. Pediatrics**	443 (10.3)
** 5. Internal medicine**	1421 (32.9)
** 6. Cardiothoracic**	234 (5.4)
**Symptoms**	
** 1. No COVID-19 related symptoms**	3939 (91.3)
** 2. Fever**	262 (6.1)
** 3. Cough**	165 (3.8)
** 4. Diarrhea**	85 (2.0)
** 5. Sore throat**	106 (2.5)
** 6. Vascular event**	44 (1.0)
**Morbidities**	
** 1. (N = 3659)**	298 (8.1)
** 2. HTN (N = 3659)**	352 (9.6)
**No. of PCR done**	4008 (92.9)
**No. of AB assay done**	2951 (68.4)

The unadjusted positivity rate of SARS-CoV-2 PCR during the study period was 154 (3.84%; 95% CI 3.29–4.48), while that of SARS-CoV-2 antibodies in the negative PCR group was 877 (29.96%; 95% CI 28.33%–31.65%) during the same period. With adjustment for test sensitivity and specificity, the positive PCR rate ranged from 3.94% in the high sensitivity scenario (95% CI: 3.34–4.62) to 5.28% (95% CI: 4.47–6.18) in the low sensitivity scenario. The adjusted SARS-CoV-2 antibody seroprevalence was 29.82 (95% CI: 28.16–31.51) (Table 2).

**Table 2:** Results of SARS-CoV-2 screening by PCR and total antibody.

	No. (unadjusted %, 95% CI)	Adjusted * % (95% CI)
**Positive PCR in total group (N = 4008)**	154 (3.84, 3.29–4.48)	Scenario1 (sensitivity 71%)
		5.28 (4.47–6.18)
		Scenario 2 (sensitivity 95%)
		3.94 (3.34–4.62)
Positive Antibody among negative PCR group (N = 2927**)	877(29.96, 28.31–31.66)	29.82 (28.16–31.51)
Negative PCR and Negative AB (N = 2927*)	1927(65.84, 64,1–67.53)	
Positive PCR and Negative AB (N = 2927*)	55 (1.88, 1.45–2.44)	
Positive PCR and positive AB (N = 2927*)	68 (2.32, 1.84–2.94)	

* Adjustment for sensitivity and specificity of the test.

**The total number of subjects with both tests (PCR and total AB) determined.

**Fig 1:**
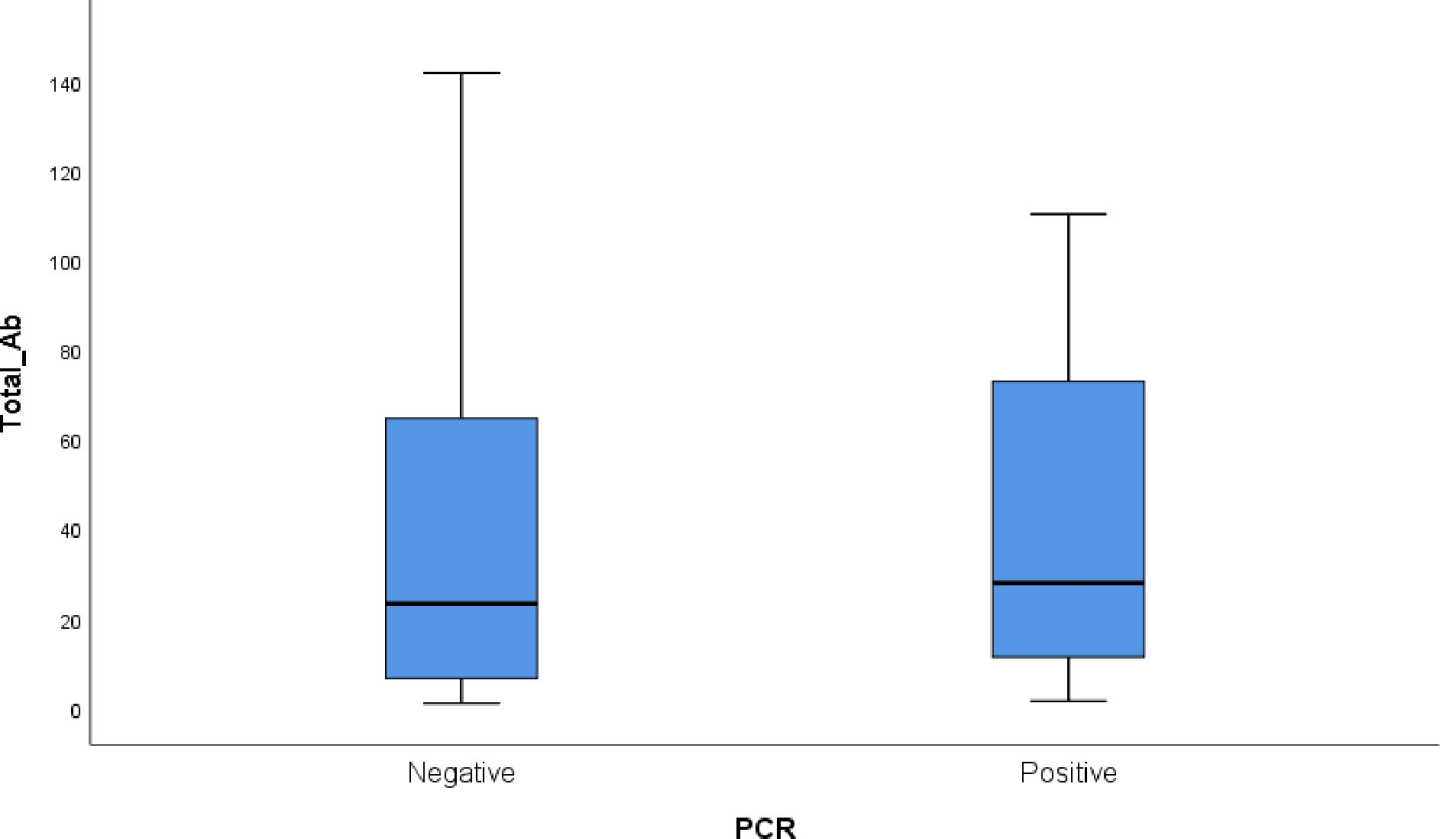
Box plot of antibody level in the positively tested antibody group.

Among the positive antibody group, the level of antibodies did not show any statistical difference between the negative and positive PCR subjects. The median and IQR of SARS-CoV-2 antibodies among the PCR positive group was 26.6 (11.90–68.40) versus 23.70 (6.60–65.60) among the PCR negative group (P value = 0.11) (Fig 1).

Males showed a higher risk for getting the COVID-19 infection as detected by positive PCR (OR adjusted for age was 1.45, 95% CI 1.06–1.98). On analyzing the adult group separately for comorbid conditions, males preserved their risk differential after adjustment (diabetes and hypertension). Age and comorbid conditions did not show any significant relation to PCR positivity rate (Table 3).

**Table 3:** Epidemiological profile of SARS-C-V-2 PCR positive and antibody seropositive subjects.

	Total no.	PCR +ve No. (%)	Adjusted OR (95% CI)	Total no.	AB +ve* No. (%)	Adjusted OR (95% CI)
All age groups						
**Age (years)**					
<18	841	25 (3.0)	1	642	135 (21.0)	1
18–	1268	43(3.4)	1.067(0.66–1.73)	923	361 (39.1)	2.19 (1.73–2.77)
40–	1136	54(4.8)	1.39 (0.87–2.26)	768	273 (35.5)	1.94 (1.52–2.47)
≥60	763	32(4.2)	1.55 (0.933–2.62)	471	108 (22.9)	1.05 (0.79–1.40)
**Gender**						
Male	1732	76 (4.4)	1.45(1.06–1.98)	1258	313 (24.9)	0.63(0.53–0.75)
Female	2276	78 (3.4)	1	1546	564 (36.5)	1
Adult Group (>18 years)						
**Age (years)**					
18–	1268	43(3.4)	1	923	361 (39.1)	1
40–	1136	54(4.8)	1.42 (0.89–2.27)	768	273 (35.5)	0.93 (0.75–1.16)
≥60	763	32(4.2)	1.2 (0.73–2.01)	471	108 (22.9)	0.48(0.37–0.77)
**Gender**						
Male	1255	68 (5.4)	1.63 (1.09–2.43)	878	241(27.4)	0.63 (0.52–0.77)
Female	1912	61 (3.2)	1	1284	501 (39)	1
**DM**						
Negative	2470	93 (3.8)	1	1794	597 (33.3)	1
Positive	265	9 (3.4)	1.19(0.56–2.52)	142	44 (31.0)	1.03 (0.7–1.56)
**HTN**						
Negative	2398	95 (4.0)	1	1732	579 (33.4)	1
Positive	337	7(2.1)	0.47 (0.20–1.08)	204	62 (30.4)	0.92 (0.65–1.31)

* The percentage is calculated among the PCR-ve group.

Regarding the seroprevalence of SARS-CoV-2 antibodies, the adult and middle-age group had significantly higher seroprevalence rates when compared to the younger age group of less than 18 years (39% and 35% versus 21%, respectively), while males showed lower seroprevalence rates when compared to females (24.9% versus 36.5%). The effect of gender remained significant in the adult group analysis after adjustment for age and comorbid conditions. The odds ratio of male gender was 0.63 (95% CI: 0.52–0.77) after adjustment for age and comorbid conditions (Table 3).

The older age group (over 60 years) had lower seroprevalence rates when compared to the adult group (from 18 to less than 40) (22.9 versus 39.1, respectively) with an odds ratio of 0.48 (95% CI 0.37–0.77), adjusted for gender and comorbid conditions. Diabetic and hypertensive subjects showed comparable seroprevalence rates to those of non-diseased subjects.

## Discussion

### PCR detection rate

The PCR detection rate in the study group was 3.84%. Most estimations of the disease incidence in various countries are based on the vigorous surveillance system [16]. In Egypt, the reported cases are consensually believed to be highly underreported [17]. This study adds insight on the number of active cases in Cairo, one of the highest density population areas. Although the frequency of infected cases in the community varies geographically as well temporally, the findings of this research revealed relatively higher rates when compared to other published figures. Reported prevalence rates in Italy of SARS-CoV-19 by PCR were 2.6% at the start of the lockdown, with a comparable rate (2.5%) in Sweden. The PCR detection rate was reported as less than 1% in Iceland [18]. Given the limitation of this hospital-based study and possible preferential testing, these findings still support wide community transmission in Cairo before the second wave of the epidemic.

### Seroprevalence of anti-SARS-CoV-2 antibodies

Epidemiological data of SARS-CoV-2 are mostly restricted to laboratory-confirmed cases for symptomatic patients. Conversely, the SARS-CoV-2 infection can present as an asymptomatic or mild disease in major sections of the population that do not seek medical advice. Therefore, the actual burden of SARS-CoV-2 can be minimized. Improved serological detection of specific SARS-CoV-2 antibodies could help calculate the true numbers of infections and enhance the understanding of related epidemiology [19–21].

Among the 2,927 subjects tested for both PCR and SARS-CoV-2 antibodies, 877 subjects (almost 30%) tested positive for antibodies with negative PCR (95% CI 28.33–31.65), denoting a past infection of SARS-COV-2 in previous months.

The literature shows that SARS-CoV-2 seroprevalence varies markedly, as expected, among geographic regions, which is sensibly elucidated by the variation in the community transmission of the infection. The results of the current study revealed a seroprevalence rate of 30%. The published data in the US show seroprevalence that ranges from less than 1% to 23% [22]. In Europe, reported seroprevalence rates have varied among different countries, with about 3.4% in Demark, 5% in Spain, and up to 23% in some areas of Italy [23–25]. An earlier study reported a seroprevalence of 17% in Iran [26].

Once more, the seroprevalence results underscore the high transmission of the infection in the community.

The timing of the study may be related to the observed high seroprevalence rates. The present study measured the seroprevalence at the end of wave 1 of the epidemic and may really reflect the cumulative infection rate in the community in contrast to many studies that measured it at the beginning or in the middle of the first wave.

This implies that the infection may be much more commonly spread than is indicated by the number of confirmed cases. Other seroprevalence studies have been directed in various territories of the world, demonstrating that for each reported case, the genuine number of diseases in the population is higher [27–30]. The discrepancy between the PCR positive cases and negative antibodies is expected as antibodies usually take 7–10 days and can even become detectable by the third week. By that time, the patient’s infectiousness begins to decline, and PCR may turn negative. The antibodies determine whether the patient has previously been exposed to infection but cannot be used to assess their current infection status. Adding to this, about 33% of COVID-19 patients were found to have no detectable antibodies.

### Factors associated with infection and seroprevalence

The present study showed that males had higher PCR detection rates in contrast to females who showed higher seroprevalence rate of anti-SARS-CoV-2 antibodies. These differential findings are not supported consistently in previous research. Early epidemiological studies in China, India, and Iran indicated that SARS-CoV2 infected fewer females [31–37]. Females may be less vulnerable to SARS-CoV-2 infection and/or less likely to show signs of COVID-19, according to these findings. However, with the rapid spread of SARS-CoV-2 across the world and the rise in epidemiological research, more recent studies have found no substantial differences in COVID-19 incidence between men and women [38]. On the contrary, female patients have better outcomes than male patients, according to several reports [39–41].

A point to mention is that this study was carried out around the peak of the first wave and the following 3 months, compared to other studies that were carried out earlier during the first wave of the epidemic. Clearance of antibodies is a point to be further investigated if it has a longer duration in females.

Although the mechanisms underlying sex-specific COVID-19 outcomes are unclear, a complex interaction of physiological, behavioral, ecological, and sociocultural influences is likely to be involved. There have been reports of sex differences in immune responses to infectious diseases, as well as the role of sex steroids in immunity [39, 42]. Estrogen can protect against COVID-19, according to some researchers [42–44].

Hypertension and diabetes failed to show any relation with either infection or seroprevalence. The relation of diabetes to infection and seroprevalence is controversial. There is wide acceptance that diabetes increases the severity of and mortality from COVID-19 [45, 46]. On the other hand, little published research has highlighted the risk of infection of SARS-CoV-2 among diabetics [47, 48]. Although there are some hints of increased susceptibility to infection among diabetics, the findings are inconsistent, with some research pointing to a similar prevalence of diabetes in COVID-19 patients to that in the overall population, suggesting no relation of diabetes to susceptibility of the infection [49, 50]. Hypertension is another non-communicable disease that has been linked to the severity and fatality of COVID-19, but its relation to the infection risk is lagging [51]. One limitation of this study is that it depended on self-reporting of hypertension and diabetes.

The younger age group (less than 18 years) expressed the least PCR positivity rate and the least seroprevalence rate (3% and 21%, respectively). This observed difference between the different age groups was not statistically significant in the positivity rate but was in the seroprevalence analysis. These findings of seroprevalence rates are in line with another research [25, 27, 29, 52].

The peak of the first wave in Egypt was in June 2020, which corresponded with the highest PCR and positive antibody detection in the study sample. The seroprevalence rate showed a decline in subsequent months, which is aligned with other studies. Röltgen et al. showed that outpatient and asymptomatic individuals’ SARS-CoV-2 antibodies, including IgG, progressively decreased during observation up to 5 months post-infection [53]. Findings from some research propose a weaker immune response to SARS-CoV-2 infection in asymptomatic individuals and state that the antibody level begins to decrease within 2–3 months after infection [54, 55]. Wang et al. also concluded that the antibody level was highest during the 31–40 days since onset and then decreased slightly [56].

#### Study limitations

This study was carried out for patients attending ASU seeking hospital services, which makes the sample not fully representative of the Cairo population. The laboratory tests were not done for all patients for sampling problems, unavailability of certain kits, or laboratory errors. Some data on hypertension and diabetes were based on participant’s self-reporting.

## Conclusion

A total of 4,313 subjects were enrolled in our study. SARS-CoV-2 PCR was 3.84%, and SARS-CoV-2 antibody seroprevalence was 29.82%, which highlights a high burden of infection in the community. Males had higher PCR detection rates in contrast to females, who showed higher seroprevalence rate of anti-SARS-CoV-2 antibodies. The younger age group (less than 18 years) expressed the least PCR positivity rate and the least seroprevalence rate. Expanding testing of persons without symptoms would be valuable in reducing the silent spread of SARS-CoV-2 in healthcare facilities. This study results emphasize the importance of continuing public health prevention steps, such as the use of face masks and social distancing, to keep SARS-CoV-2 from spreading.

## Supporting information

S1 Data (XLSX)

S1 File (DOCX)

S2 File (DOCX)
