# Supplementary material for: SARS-CoV-2 PCR positivity rate and seroprevalence of related antibodies among a sample of patients in Cairo: Pre-wave 2 results of a screening program in a university hospital
Source: PLoS One. 2021 Jul 15;16(7):e0254581. doi: 10.1371/journal.pone.0254581 (PMC8282003; doi:10.1371/journal.pone.0254581)
Supplement: S1 File — (DOCX) [file pone.0254581.s002.docx]

**موافقة على الاشتراك فى البحث العلمى الخاص بفحص المرضى المترددين على مستشفيات جامعة عين شمس**

تهدف هذه الدراسة إلى فحص جميع المرضى المترددين علىً مستشفيات جامعه عين شمس لفيروس كورونا المستجد وذلك لمعرفه مدى انتشار المرض و ضمان سلامه المرضى بالمستشفيات.

يتم الفحص بواسطه مسحه من الأنف والفم وكذلك عينه دم صغيره وهى طرق بسيطه لا تسبب ايه أعراض جانبيه

تظهر نتيجه التحاليل في خلال يومين وسيتم إعلامك بالنتيجة .

سيتم إعلام وزاره الصحه المصريه بالنتيجة اذا كانت ايجابيه وذلك حتى توفر لك العلاج المناسب للحاله في مكان ومستشفى مناسب

لك الحق فى الرفض اوً الانسحاب من البحث

جميع البيانات الخاصه بالبحث هي لأغراض البحث العملى فقط ويتم تداولها بسريه .

□أوافق □لا أوافق على الاشتراك في الدراسة وقد تم أتاحه الوقت لى لأيه استفسارات اوً اسئله وتم الاجابه عليها

كما □أوافق □لا أوافق على حفظ العينات الخاصه بى لاستعمالها لاحقا في ايه أبحاث أخرى مستقبلية

الاسم: _______________ التوقيع: ________________ الرقم القومى:________________

This study aims to testing all patients in Ain Shams University hospitals for the Corona virus to know the extent of infection and assure safety for patients in hospital.

The test is done through a swab from nose and mouth and a little blood sample. These methods are very simple and don’t cause any side effects. The results of the test are known within two days and you will be notified accordingly.

The Ministry of Health will be notified if the result turned positive to provide you the necessary treatment and suitable treating hospital.

You have the right to refuse or withdraw from the study.

All data related to this research are used for research purpose only and managed with confidentiality.

I hereby □accept □don’t accept to participate in this study and I have been given the time for any inquiries or questions that have been answered

I hereby □accept □don’t accept to preserve samples taken from me to be used in future researches

Name __________________ Signature __________________ ID _______________
