# Supplementary material for: SARS-CoV-2 PCR positivity rate and seroprevalence of related antibodies among a sample of patients in Cairo: Pre-wave 2 results of a screening program in a university hospital
Source: PLoS One. 2021 Jul 15;16(7):e0254581. doi: 10.1371/journal.pone.0254581 (PMC8282003; doi:10.1371/journal.pone.0254581)
Supplement: S2 File — (DOCX) [file pone.0254581.s003.docx]

**فحص المرضى المترددين على مستشفيات جامعة عين شمس**

| اسم المستشفى: |  | | | | | | |  | | | | | | | | | | | | | |  | |  |
| --- | --- | --- | --- | --- | --- | --- | --- | --- | --- | --- | --- | --- | --- | --- | --- | --- | --- | --- | --- | --- | --- | --- | --- | --- |
|  |  | | | | | | |  | | | | | | | | | | | | | |  | |  |
| الاسم: |  | | | | | | |  | | | | | | | | | | | | | |  | |  |
|  |  | | | | | | |  | | | | | | | | | | | | | |  | |  |
| الرقم القومي: |  |  |  |  |  |  |  | |  |  |  | |  |  |  |  | |  | | | | |  | |
|  |  | | | | | | |  | | | | | | | | | | | | | |  | |  |
| رقم التليفون: |  | | | | | | | رقم المحمول: | | | | | | | | | | | | | |  | |  |
|  |  | | | | | | |  | | | | | | | | | | | | | |  | |  |
| عنوان السكن: |  | | | | | | | | | | | | | | | | | | |  | |  | |  |
|  |  | | | | | | | | | | | | | | | | | | |  | |  | |  |
| اسم القريب:. |  | | | | | | | | | | | | | | | | نوع القرابه: | | | | |  | |  |
|  |  | | | | | | |  | | | | | | | | | | | | | |  | |  |
|  | تليفون القريب: | | | | | | |  | | | | | | | | | | |  | | | | |  |
|  |  | | | | | | |  | | | | | | | | | | | | | |  | |  |
| تاريخ الميلاد: | .. / .. / .... | | | | | | | السن: | | | | - - سنة | | | |  |  |  |  |  |  |  |  |  |
|  |  | | | | | | |  | | | | | | | | | | | | | |  | |  |
| النوع: | □ذكر □أنثى | | | | | | |  | | | | | | | | | | | | | |  | |  |
|  |  | | | | | | |  | | | | | | | | | | | | | |  | |  |
| العمل: |  | | | | | | | | | | | | | | | | | | | | |  | |  |
|  |  | | | | | | | | | | | | | | | | | | | | |  | |  |
| هل تعانى من : | - ارتفاع في درجه الحرارة درجة الحرارة:______ | | | | | | | | | | | | | | | | | | | | |  | |  |
|  | - نهجان / ضيق بالنفس | | | | | | | | | | | | | | | | | | | | |  | |  |
|  | - حرقان بالحلق | | | | | | | | | | | | | | | | | | | | |  | |  |
|  | - كحة | | | | | | | | | | | | | | | | | | | | |  | |  |
|  | - إسهال | | | | | | | | | | | | | | | | | | | | |  | |  |
|  | - مخالط لحاله ايجابيه لفيروس كورونا المستجد | | | | | | | | | | | | | | | | | | | | |  | |  |
| هل تعانى من | - ضغط دم مرتفع - مرض السكر | | | | | | | | | | | | | | | | | | | |  |  | |  |
|  |  | | | | | | | | | | | | | | | | | | | |  |  | |  |
| نتائج التحاليل | PCR : □+veإيجابى □-ve سلبى | | | | | | | | | | | | | | | | | | | |  |  | |  |
|  |  | | | | | | | | | | | | | | | | | | | |  |  | |  |
|  | **Antibody** Titre: □+veإيجابى □-veسلبى | | | | | | | | | | | | | | | | | | | |  |  | |  |

**Examination of patients attending Ain Shams University hospitals**

**Name of the hospital**: ………………………………………………

**Patient’s name:** ……………………………………………………………..

**National ID:**

|  |  |  |  |  |  |  |  |  |  |  |  |
| --- | --- | --- | --- | --- | --- | --- | --- | --- | --- | --- | --- |

**Phone number:**

**Mobile number:**

**Home Address:**

**Relative’s name:**

**Kinship Type:**

**Relative’s Phone**:

**Date of birth**: .. / .. / ....

**Age:** - - year

**Gender:** □ Male □ Female

**Occupation:** …………………………………………………….

**Do you suffer from?**

- High temperature temperature: ______
- Shortness of breath
- Burning sensation in the throat
- Cough
- Diarrhea
- Contact for a positive case for the emerging corona virus

**Do you suffer from?**

- High blood pressure
- Diabetes

**Lab results:**

- PCR: □ positive □ negative
- Antibody Titer: ____________□ positive □ negative
