# Supplementary material for: SARS-CoV-2 PCR positivity rate and seroprevalence of related antibodies among a sample of patients in Cairo: Pre-wave 2 results of a screening program in a university hospital
Source: PLoS One. 2021 Jul 15;16(7):e0254581. doi: 10.1371/journal.pone.0254581 (PMC8282003; doi:10.1371/journal.pone.0254581)
Supplement: Attachment — Submitted filename: PONE-D-21-06534_reviewer_AAS.pdf [file pone.0254581.s004.pdf]

PLOS ONE

SARS-CoV-2 PCR positivity rate and seroprevalence of related antibodies among a sample of patients in Cairo: Pre-wave 2 results of a screening program in a university hospital

--Manuscript Draft--

Manuscript Number:	PONE-D-21-06534
Article Type:	Research Article
Full Title:	SARS-CoV-2 PCR positivity rate and seroprevalence of related antibodies among a sample of patients in Cairo: Pre-wave 2 results of a screening program in a university hospital
Short Title:	SARS-CoV-2 PCR positivity rate and seroprevalence of related antibodies among a sample of patients in Cairo.
Corresponding Author:	Isis M Mossad<br>Ain Shams University Faculty of Medicine<br>cairo, EGYPT
Keywords:	Covid-19, SARS-COV-2, Screening, PCR, Seroprevalence, Cairo.
Abstract:	Background<br><br>On 1st of December 2020 CDC Guidance for Expanded Screening Testing to Reduce Silent Spread of SARS-CoV-2 clarified that asymptomatic and pre-symptomatic infections are important contributors to the transmission of SARS-CoV-2 to the population. This is a great challenge specially in hospital setting, as nosocomial transmission of COVID-19 to healthcare personnel and other patients can have serious impacts on hospital performance. The aim of this study is to determine the SARS-CoV-2 PCR positivity rate as well the seroprevalence of the SARS-CoV-2 antibodies before the ultimate development of second wave of the epidemic in Cairo, Egypt.<br><br>Methods<br><br>our study was carried out between May 5th and end of October 2020, included All patients needing admission in Ain-Shams University Hospitals. Data collected by using interview questionnaire about demographic and clinical data, laboratory Tests included (RT-PCR) and total antibody assay for SARS-CoV-2 were done for all participants.<br><br>Results<br><br>A total of 4313 subjects were enrolled in our study (56%) were Females, Adults and middle age represented around 60%, and 91.3% did not complain of any related COVID-19 symptoms. The positivity rate of SARS-Cov-2 PCR was 3.84% (95% CI 3.29-4.48), and the SARS-CoV-2 antibody seroprevalence was 29.82 (95% CI: 28.16-31.51). Males showed higher risk for getting the COVID-19 infection, while middle age group had significantly higher antibodies seroprevalence rate.<br><br>Conclusions<br><br>expanding testing of persons without symptoms should be adopted to reduce silent spread of SARS-CoV-2 in the health care facilities. Setting prioritization criteria for screening is mandatory to overcome insufficient resources.
Order of Authors:	Samia A. Girgis
	Hala M Hafez
	Hoda Ezz Elarab
	Basma Sherif
	Moshira H Sabry
	Iman Afifi

Powered by Editorial Manager® and ProduXion Manager® from Aries Systems Corporation

	Fatma Elzahraa Hassan
	Amira Reda
	Shaimaa Elsayed
	Asmaa Mahmoud
	Petra Habeb
	Ihab S. Habil
	Rahsa S Hussein
	Isis M Mossad
	Ossama Mansour
	Ashraf Omar
	Ayman M. Saleh
	Mahmoud El-Meteini
**Additional Information:**	
**Question**	**Response**
**Financial Disclosure**<br><br>Enter a financial disclosure statement that describes the sources of funding for the work included in this submission. Review the submission guidelines for detailed requirements. View published research articles from *PLOS ONE* for specific examples.<br><br>This statement is required for submission and **will appear in the published article** if the submission is accepted. Please make sure it is accurate.	the authors didn't receive any specific funding for their work.

**Unfunded studies**<br>Enter: *The author(s) received no specific funding for this work.*<br><br>**Funded studies**<br>Enter a statement with the following details:<br>• Initials of the authors who received each award<br>• Grant numbers awarded to each author<br>• The full name of each funder<br>• URL of each funder website<br>• Did the sponsors or funders play any role in the study design, data collection and analysis, decision to publish, or preparation of the manuscript?<br>• **NO** - Include this sentence at the end of your statement: *The funders had no role in study design, data collection and analysis, decision to publish, or preparation of the manuscript.*<br>• **YES** - Specify the role(s) played.<br><br>* typeset	
**Competing Interests**<br><br>Use the instructions below to enter a competing interest statement for this submission. On behalf of all authors, disclose any competing interests that could be perceived to bias this work—acknowledging all financial support and any other relevant financial or non-financial competing interests.<br><br>This statement is required for submission and **will appear in the published article** if the submission is accepted. Please make sure it is accurate and that any funding sources listed in your Funding Information later in the submission form are also declared in your Financial Disclosure statement.<br><br>View published research articles from *PLOS ONE* for specific examples.	The authors have declared that no competing interests exist.

Powered by Editorial Manager® and ProduXion Manager® from Aries Systems Corporation

**NO authors have competing interests**<br><br>Enter: *The authors have declared that no competing interests exist.*<br><br>**Authors with competing interests**<br><br>Enter competing interest details beginning with this statement:<br><br>*I have read the journal's policy and the authors of this manuscript have the following competing interests: [insert competing interests here]*<br><br>* typeset	
**Ethics Statement**<br><br>Enter an ethics statement for this submission. This statement is required if the study involved:<br><br>• Human participants<br>• Human specimens or tissue<br>• Vertebrate animals or cephalopods<br>• Vertebrate embryos or tissues<br>• Field research<br><br>Write "N/A" if the submission does not require an ethics statement.<br><br>General guidance is provided below. Consult the submission guidelines for detailed instructions. **Make sure that all information entered here is included in the Methods section of the manuscript.**	The research was approved by the University Ethical Research Committee.<br>FW A00017585 assurance number, Positive cases were reported to the Ministry of Health and Population (MOHP). The guidelines of isolation and treatment protocol of MOHP were followed.

Powered by Editorial Manager® and ProduXion Manager® from Aries Systems Corporation

**Format for specific study types**<br><br>**Human Subject Research (involving human participants and/or tissue)**<br>• Give the name of the institutional review board or ethics committee that approved the study<br>• Include the approval number and/or a statement indicating approval of this research<br>• Indicate the form of consent obtained (written/oral) or the reason that consent was not obtained (e.g. the data were analyzed anonymously)<br><br>**Animal Research (involving vertebrate animals, embryos or tissues)**<br>• Provide the name of the Institutional Animal Care and Use Committee (IACUC) or other relevant ethics board that reviewed the study protocol, and indicate whether they approved this research or granted a formal waiver of ethical approval<br>• Include an approval number if one was obtained<br>• If the study involved *non-human primates*, add *additional details* about animal welfare and steps taken to ameliorate suffering<br>• If anesthesia, euthanasia, or any kind of animal sacrifice is part of the study, include briefly which substances and/or methods were applied<br><br>**Field Research**<br><br>Include the following details if this study involves the collection of plant, animal, or other materials from a natural setting:<br>• Field permit number<br>• Name of the institution or relevant body that granted permission	
**Data Availability**<br><br>Authors are required to make all data underlying the findings described fully available, without restriction, and from the time of publication. PLOS allows rare exceptions to address legal and ethical concerns. See the PLOS Data Policy and FAQ for detailed information.	Yes - all data are fully available without restriction

A Data Availability Statement describing where the data can be found is required at submission. Your answers to this question constitute the Data Availability Statement and **will be published in the article**, if accepted.<br><br>**Important:** Stating 'data available on request from the author' is not sufficient. If your data are only available upon request, select 'No' for the first question and explain your exceptional situation in the text box.<br><br>Do the authors confirm that all data underlying the findings described in their manuscript are fully available without restriction?	
**Describe where the data may be found in full sentences. If you are copying our sample text, replace any instances of XXX with the appropriate details.**<br><br>• If the data are **held or will be held in a public repository**, include URLs, accession numbers or DOIs. If this information will only be available after acceptance, indicate this by ticking the box below. For example: *All XXX files are available from the XXX database (accession number(s) XXX, XXX.).*<br>• If the data are all contained **within the manuscript and/or Supporting Information files**, enter the following: *All relevant data are within the manuscript and its Supporting Information files.*<br>• If neither of these applies but you are able to provide **details of access elsewhere**, with or without limitations, please do so. For example:<br><br>*Data cannot be shared publicly because of [XXX]. Data are available from the XXX Institutional Data Access / Ethics Committee (contact via XXX) for researchers who meet the criteria for access to confidential data.*<br><br>*The data underlying the results presented in the study are available from (include the name of the third party*	The data underlying the results presented in the study are available upon reasonable request from authors after consulting the Ethical committee

and contact information or URL).<br>• This text is appropriate if the data are owned by a third party and authors do not have permission to share the data.<br><br>* typeset	
Additional data availability information:	

Manuscript Click here to access/download;Manuscript;Manuscript.docx

SARS-CoV-2 PCR positivity rate and seroprevalence of related antibodies among a sample of patients in Cairo: Pre-wave 2 results of a screening program in a university hospital

Samia A. Girgis[1], M.D, Hala M Hafez[2], M.D, Hoda Ezz Elarab[3], M.D, Basma Sherif [3], M.D, Moshira H Sabry[4], M.D, Iman Afifi[4], M.D, Fatma Elzahraa Hassan[4], M.Sc, Amira Reda[4], MBBCH , Shaimaa Elsayed[4] , MBBCH, Asmaa Mahmoud[4], M.Sc, Petra Habeb[4], M.Sc, Ihab S. Habil[5], M.D, Rahsa S Hussein[5] , M.D, Isis M Mossad[5], M.D, Ossama Mansour[6], M.D, Ashraf Omar[7], M.D, Ayman M. Saleh[8], M.D, Mahmoud El-Meteini[9], M.D.

[1]Professor of Clinical Pathology, Head of Infection Control Unit and Vice Director of Ain Shams University Hospitals, Cairo, Egypt

[2] Professor of Clinical Pathology, Head of Clinical Microbiology Unit, Ain Shams University Hospitals, Cairo, Egypt

[3]Department of Clinical Pathology, Faculty of Medicine, Ain Shams University, Cairo, Egypt

[4]Infection Control Unit, Ain Shams University Hospitals, Cairo, Egypt.

[5]Department of Community, Environmental and Occupational Medicine, Faculty of Medicine, Ain Shams University, Cairo, Egypt.

[5] Executive Director of Ain Shams University Hospitals, Faculty of Medicine, Ain Shams University, Cairo, Egypt.

[6]Vice Dean of Faculty of Medicine, Ain Shams University, Cairo, Egypt.

[7]*Dean of Faculty of Medicine and chairman of board of Ain Shams University Hospitals, Ain Shams University, Cairo, Egypt.*

[8]*Vice President of Ain Shams University, Cairo, Egypt.*

[9]*President of Ain-Shams University, Cairo, Egypt*

Corresponding Author: *Isis Magdy Mossad*

Address: 136 Banafseg 5, First Settlement, Cairo, Egypt.

Telephone number: 0201271010862

E-mail address: Isis.magdy@med.asu.edu.eg

Word count for the body of the text: 3428

Abstract:

Background: On 1st of December 2020 CDC Guidance for Expanded Screening Testing to Reduce Silent Spread of SARS-CoV-2 clarified that asymptomatic and pre-symptomatic infections are important contributors to the transmission of SARS-CoV-2 to the population. This is a great challenge specially in hospital setting, as nosocomial transmission of COVID-19 to healthcare personnel and other patients can have serious impacts on hospital performance. The aim of this study is to determine the SARS-CoV-2 PCR positivity rate as well the seroprevalence of the SARS-CoV-2 antibodies before the ultimate development of second wave of the epidemic in Cairo, Egypt. **Methods:** our study was carried out between May 5th and end of October 2020, included All patients needing admission in Ain-Shams University Hospitals. Data collected by

using interview questionnaire about demographic and clinical data, laboratory Tests included (RT-PCR) and total antibody assay for SARS-CoV-2 were done for all participants. **Results**: A total of 4313 subjects were enrolled in our ~~study (56%) were Females~~, Adults and middle age represented around 60%, and 91.3% did not complain of any related COVID-19 symptoms. The positivity rate of SARS-Cov-2 PCR was 3.84 % (95% CI 3.29-4.48), and the SARS-CoV-2 antibody seroprevalence was 29.82 (95% CI: 28.16-31.51). Males showed higher risk for getting the COVID-19 infection, while middle age group had significantly higher antibodies seroprevalence rate. **Conclusions:** expanding testing of persons without symptoms should be adopted to reduce silent spread of SARS-CoV-2 in ~~the~~ health care facilities. Setting prioritization criteria for screening is mandatory to overcome insufficient resources.

Introduction

The new coronavirus, officially named severe acute respiratory syndrome coronavirus 2 (SARS-CoV-2) infected around eighteen million worldwide by the start of August 2020, with almost 700 thousand deaths (*1*) and the numbers are increasing every day in a steep manner. In Egypt, more than 4800 deaths and almost 94 thousand infections were reported by that time.(*2*) The capital, Cairo, with its high-density population took the lead among Egyptian governorates in terms of the number of COVID-19 cases, according to data declared by the Egyptian Ministry of health (3). However, the true prevalence, that is, the number of people who are currently infected or have been infected by SARS-COV-2 over the entire population is likely much higher, and accordingly the mortality rate should be fundamentally lower. The COVID-19 surveillance in Egypt depends mainly on the reported PCR detection, that is usually carried out in symptomatic cases. The total number of screening tests done in Egypt has been substantially lower than that done in other countries (4)

Studies show that about 30%–60% of COVID-19 patients have mild or no symptoms and still have the ability to spread the infection. (5) Previous studies have suggested that only a small fraction of asymptomatic persons may eventually develop symptoms.(6) (7)(8) These facts add to the struggle of estimating the magnitude of the COVID-19 in a community.

Many ~~Researchers is~~ attempting to estimate the rates of infection in the community through epidemiological models(9) (10) , or structural assumptions (11). With limited testing availability

and high proportion of mild and asymptomatic infections, there is under-ascertainment of SARS-CoV-2 infections through passive case reporting. (6) (7)(8) In such case, seroprevalence surveys of SARS-CoV-2 antibodies are important for refining estimates of infection and transmission (12) Moreover, seroprevalence studies can give information on risk factors for the disease, such as a patient's age, location, or underlying health conditions. Furthermore, they could show significant medical data on immune reactions to the virus, as duration antibodies in patients after infection.(13).

In hospital setting, The SARS-CoV-2 infection presents a great challenge where it is highly infectious during the pre-symptomatic period in patients. The nosocomial transmission of COVID-19 to health-care workers and other patients can have serious impacts on hospital operation, including the suspension of new admissions and the closing of hospital wards. Pre-admission screening by PCR is a policy recommended by different agencies including the Centers for Disease Control and Prevention in the USA and the Public Health England guidelines depending on testing capacity and disease prevalence.

By the beginning of May 2020, the Ain Shams University (ASU) hospitals in Cairo have adopted a universal screening program for all patients requiring admission in the hospitals. The Screening process included PCR testing and total antibody assay before admission.

With scarce data available on the epidemiology of the COVID-19 in Egypt, the aim of the current research is to determine the SARS-CoV-2 PCR positivity rate as well the seroprevalence of the

SARS-CoV-2 antibodies before the ultimate development of potential second wave of the epidemic in Cairo, Egypt. The findings were based on results of a universal screening program for patients in Ain Shams University hospitals in Cairo.

Subjects and methods

The current study was carried out between May 5th and end of October 2020.

Study setting: This study took place in Ain-Shams University Hospitals. It is a campus including the following main hospitals: Gynecology and Obstetrics, Oncology, Pediatrics, Psychiatry, Internal Medicine, surgical, cardiothoracic and Geriatrics.

Study population: All patients needing admission in Ain-Shams University Hospitals were eligible for the study.

The hospital screening program: By the beginning of the epidemic in Egypt, Ain Shams University (ASU) hospitals established a symptom- based screening clinic for all patients seeking hospital services. SARS-CoV-2 PCR and total antibody assay were done for all patients needing hospitalization.

Study methods:

Every enrolled patient was subjected to:

1) An interview questionnaire including personal data (age, gender, residence, contact details, history of contact to a COVID-19 case), clinical data history (Fever, Cough, sore throat), comorbidities (Diabetes, Hypertension)

2) Laboratory Tests: Reverse Transcription Polymerase Chain Reaction (RT-PCR) and total antibody assay for SARS-CoV-2

Specimen collection and handling:

1- Combined nasopharyngeal and oropharyngeal swabs were collected from enrolled patients using sterile swabs with synthetic tips (nylon or dacron) and flexible, plastic shafts as recommended by the US Center for Disease Control and Prevention (CDC).

First, the swab was inserted into the posterior oropharynx and rubbed against the posterior pharyngeal wall and tonsillar pillars avoiding the tongue, teeth and gums. Then, the patient’s head was tilted 70 degrees and the same swab was inserted slowly through the patient’s nostril parallel to the palate until resistance is encountered. The swab should reach a depth equivalent to the distance between the patient’s nostril and the tragus of the ear. The swab was left in place for few seconds for enabling secretion absorption and then removed slowly while rotating it. Finally, the swab was immediately inoculated into a sterile tube, containing 2 mL of transport media and transported immediately to the laboratory at a temperature 2-8°C.

2- Serum samples: 3 ml whole blood sample was collected from each patient by peripheral venipuncture on a clot activator and gel separator vaccutainer tube. The tubes were immediately centrifuged and separated serum was used to measure the SARS-COV2 total antibodies using the Elecsys® Anti-SARS-CoV-2 immunoassay (Roche).

I-Detection of SARS-COV2 RNA by Reverse Transcription Real Time Polymerase Chain Reaction (rRT-PCR):

Nucleic acid extraction:

Viral RNA was extracted using the Viasure RNA/DNA extraction kit (*CerTest Biotec*, Spain) as follows:

- Each sample was mixed well by gentle vortexing and a volume of 300ul was transferred into a deep-well plate.
- A total volume of 314ul lysing solution containing 300ul of lysis buffer, 10ul proteinase K solution and 4ul of carrier RNA was added into each sample well.
- Sample plate was then loaded onto the Chemagic 360 automatic extractor (Perkin Elmer, Germany). Viral RNA was then automatically extracted by binding to the surface of magnetic beads. Impurities such as salts, metabolites, and soluble macromolecular cellular components were efficiently removed by a series of quick washing steps.
- Unless immediately processed, extracted RNA was kept at -20°C.

Detection of SARS-CoV2 RNA by rRT-PCR:

SARS-CoV-2 Real Time PCR Detection Kit (*CerTest Biotec*, Spain) is designed for the diagnosis of SARS-CoV-2 in respiratory samples. The detection is done in one step real time (RT) format

where the reverse transcription and the subsequent amplification of specific target sequence occur in the same reaction well. The isolated RNA target is transcribed generating complementary DNA by reverse transcriptase which is followed by the amplification of a conserved region of ***ORF1ab*** and ***N*** genes for SARS-CoV-2 using specific primers and a fluorescent-labeled probe.

~~The SARS-CoV-2 Real Time PCR Detection Kit is based on the 5´ exonuclease activity of DNA polymerase. During DNA amplification, this enzyme cleaves the probe bounded to the complementary DNA sequence, separating the quencher dye from the reporter. This reaction generates an increase in the fluorescent signal which is proportional to the quantity of target template. This fluorescence can be measured on Real Time PCR platforms.~~

<u>The amplification protocol:</u>

Program the thermocycler following the conditions listed below and start the run:

**Cycles**	**Step**	**Time**	**Temperature**
1	Reverse Transcription	15 min	45℃
1	Initial denaturation	2 min	95℃
45	Denaturation	10 ~~seg~~	95℃
	Annealing/Extension (Data collection)	50 ~~seg~~	60℃

Quality Control:

The *SARS-CoV-2* Real Time PCR Detection Kit contains a positive and a negative control that were included in each run to correctly interpret the results. Also, the internal control (IC) in each well confirms the correct performance of the technique.

Interpretation of Test Results:

The use of positive and negative controls in each run, validate the reaction by checking the absence of signal in the negative control well and the presence of signal for SARS-CoV-2 in the positive control well. The Internal Control signal was checked to verify the correct functioning of the amplification mix. The analysis of the samples was done by the software of the used real time PCR equipment itself according to manufacturer´s instructions.

A sample was considered positive if the Ct value obtained is less than 38 and the internal control shows or not an amplification signal. Sometimes, the detection of internal control is not necessary because a high copy number of targets can cause preferential amplification of target-specific nucleic acids.

A sample was considered negative, if the sample shows no amplification signal in the detection system but the internal control is positive. An inhibition of the PCR reaction can be excluded by the amplification of internal control.

The result was considered invalid if there is signal of amplification in negative control or absence of signal in the positive well.

II- <u>Detection of SARS-COV2 Total Antibodies:</u>

The Elecsys® Anti-SARS-CoV-2 is an immunoassay for the in vitro qualitative detection of antibodies (including IgG) to SARS-CoV-2 in human serum and plasma. The assay uses a recombinant protein representing the nucleocapsid (N) antigen in a double-antigen sandwich assay format, which favors detection of high affinity antibodies against SARS-CoV-2. The test is intended as an aid in the determination of the immune reaction to SARS-CoV-2.

A volume of 20 µL of the patient serum was incubated with a mix of biotinylated and ruthenylated nucleocapsid (N) antigen. Double-antigen sandwich immune complexes are formed in the presence of corresponding antibodies. After addition of streptavidin-coated microparticles, the double-antigen complexes bind to the solid phase via interaction of biotin and streptavidin. The reagent mixture is transferred to the measuring cell, where the microparticles are magnetically captured onto the surface of the electrode. Unbound substances are subsequently removed. Electrochemiluminescence is then induced by applying a voltage and measured with a photomultiplier. The signal yield increases with the antibody titer.

A cut-off index <1.0 is considered non-reactive whereas a cut-off index ≥ 1.0 is considered reactive.

Statistical analysis:

Data were validated, cleaned, and entered in a spreadsheet. Qualitative data was presented in frequency and related percentage. Level of Antibodies was presented by median and interquartile range with Mann Whitney test used for comparison. Unadjusted frequency of positive screening among the total was calculated with 95% confidence interval. Given that SARS-CoV-2 PCR sensitivity was reported to be between 71% and 95% (14),the PCR positivity was adjusted for test sensitivity for both scenarios with specificity of 99.9%. The antibody seroprevalence was adjusted for the kit sensitivity and specificity. According to the manufacturer's package insert, Elecsys®.Anti-SARS-CoV-2 exhibits high overall clinical specificity of 99.81% with no cross-reactivity to the common cold coronaviruses and a sensitivity of 100%. We used Clopper–Pearson exact method to calculate 95% confidence intervals.

Comparison between groups was done using Chi-square test with a "P" value of 0.05 as the level of significance. Odds ratio was calculated for estimation of risk with 95% confidence interval. Logistic regression was used for adjustment of the confounding factors.

SPSS program version 15 was used for analysis. Epitools Epidemiological Calculators. Ausvet. was used for adjustment for tests' sensitivity and specificity. Available at: http://epitools.ausvet.com.au

Ethical considerations:

The protocol of the research was approved by the University Ethical Research Committee. Positive cases were reported to the Ministry of Health and Population (MOHP). The guidelines of isolation and treatment protocol of MOHP were followed.

Results

The current research enrolled 4313 subjects during the study period. A total of 4008 and 2951 patients had the PCR test and the antibody assay done, respectively. Females constituted 56% of the study sample. Adults and middle age represented around 60% of the sample. The vast majority of patients (91.3%) did not complain of any related COVID-19 symptoms.

The unadjusted positivity rate of SARS-Cov-2 PCR during the study period was 3.84 % (95% CI 3.29-4.48), while that of SARS-CoV-2 antibodies in negative PCR group was 29.96% (95% CI 28.33%-31.65%) during the same period. With adjustment for test sensitivity and specificity, the positive PCR rate ranged from 3.94% in high sensitivity scenario (95% CI: 3.34 -4.62) to 5.28% (95% CI: 4.47- 6.18) in low sensitivity scenario. The adjusted SARS-CoV-2 antibody seroprevalence was 29.82 (95% CI: 28.16-31.51). Among the positive Antibody group, the level of antibody did not show any statistical difference between the negative and positive PCR subjects.

The Median and IQR of SARS-CoV-2 antibodies among PCR positive group was 26.6(11.90-68.40) Versus 23.70 (6.60- 65.60) among PCR negative group (P value =0.11).

Males showed higher risk for getting the COVID-19 infection as detected by positive PCR (OR adjusted for age was 1.45, 95% CI 1.06 -1.98). On analyzing the adult group separately for comorbid conditions, males preserved their risk differential after adjustment for comorbid conditions (Diabetes and hypertension). Age and comorbid conditions did not show any significant relation to PCR positivity rate.

Regarding the seroprevalence of SARS-CoV-2 Antibodies, adult and middle age group had significantly higher seroprevalence rate compared to younger age group less than 18 years (39% and 35% versus 21% respectively) while males showed lower seroprevalence rate compared to females (24.9% versus 36.5%). The effect of gender remains significant in adult group analysis after adjustment for age and comorbid conditions. The odds ratio of male gender was 0.63 (95% CI: 0.52-0.77) after adjustment of age and comorbid conditions.

Old age group (more than 60 years) had lower seroprevalence rate compared to the adult group (from 18 to less than 40) (22.9 versus 39.1, respectively) with an odds ratio of 0.48(95% CI 0.37-0.77) adjusted for gender and comorbid conditions. Diabetic and hypertensive subjects showed comparable seroprevalence rates to that of non-diseased subjects.

Discussion:

PCR Detection rate

The PCR detection rate in the study group was 3.84%.Most estimation of the disease incidence in various countries is based on vigorous surveillance system(*1*) . In Egypt, the reported cases are consensually believed to be much underreported (16). This study adds insight on the number of active cases in Cairo, one of the highest density population areas. Although the frequency of infected cases in the community varies geographically as well temporally, yet the findings of this research revealed relatively higher rate compared to other published figures. In Europe, reported prevalence rates of SARS-CoV-19 by PCR was 2.6% in Italy at the start of lockdown, with comparable rate (2.5%) in Sweden. The PCR detection rate was reported to less than 1% in Iceland.(17) Given the limitation of this hospital-based study and possible preferential testing, these findings still support wide community transmission in Cairo before the second wave of the epidemic.

Seroprevalence of anti-SARS-CoV-2 antibodies

The epidemiological data of COVID-19 are mostly restricted to laboratory-confirmed cases for symptomatic patients. Conversely, the SARS-CoV-2 infection can manifest as an asymptomatic or mild disease in great sector of population that do not seek medical advice. Therefore, the true extent of the burden of COVID-19 may be underestimated. Improved serological detection of

specific antibodies against SARSCoV-2 could help estimate the true numbers of infections and improve understanding of the associated epidemiology (18)(19)(20).

Amongst 2927 subjects who were tested for both PCR and SARS-CoV-2 antibodies, 877 subjects (almost 30%) were tested positive for antibodies with negative PCR (95% CI 28.33-31.65), denoting a past infection by SARS-COV-2 in the previous months.

The literature showed that SARS-CoV-2 seroprevalence varies markedly, as expected, among geographic regions which is sensibly elucidated by the variation in the community transmission of the infection. The results of the current study revealed a seroprevalence rate of 30%. The published data in USA showed seroprevalence that ranges from less than 1% to 23% (21). In Europe, reported seroprevalence rates varied among different countries; with about 3.4% in Demark, 5% in Spain up to 23% in some areas in Italy (22) (23)(24). An earlier study reported a seroprevalence of 17% in Iran.(25)

Once more, the seroprevalence results underscored the high transmission of the infection in the community.

Timing of the study may be related to the observed high seroprevalence rate. The current study measured the seroprevalence at the end of wave 1 of the epidemic and may really reflects

cumulative infection rate in the community in contrast to many studies that measured it at the beginning or in the middle of the first wave.

This implies that the infection may be much more widespread than indicated by the number of confirmed cases. Other seroprevalence studies have been directed in various territories of the world demonstrating that for each reported case the genuine number of diseases in the population is higher (26) (27)(28)(29).

Factors associated with infection and seroprevalence

The current study showed that males had higher PCR detection rate in contrast to females who showed higher seroprevalence rate of anti-SARS-CoV-2 antibodies. These differential findings are not supported consistently in previous researches. Early epidemiological studies conducted in China, India, and Iran revealed that fewer females were infected by SARS-CoV2 (30)(31)(32)(33)(34)(35)(36). These results suggest that females may be less susceptible to SARS-CoV-2 infection and/or less likely to present symptoms of COVID-19. However, with the rapid spread of SARS-CoV-2 in the world and an increase in epidemiological studies around the globe, more recent studies found that there were no significant differences between men and women in the incidence of COVID-19 (*37*).On the other hand, many studies have reported that female patients have better outcomes than male patients (38)(39)(40)

A point to mention is that this study was carried out around the peak of the first wave and the following 3 months compared to the other studies which were carried out earlier during the first wave of the epidemic. Clearance of antibodies is a point to be furtherly investigated If it takes longer duration in females.

Although the mechanisms underlying the sex-specific COVID-19 outcomes are not entirely clear, it is possible that this involves a complex interplay among biological, behavioral, environmental, and socioeconomic factors. Sex differences in the immune response to infectious diseases and the role of sex steroids regulating immunity have been reported (41) (38). It has been proposed that estrogens may exert protective effects against COVID-19 (42)(41)(43)

Hypertension and diabetes failed to show any relation with either infection or seroprevalence. The relation of diabetes to infection and seroprevalence is controversial. There is wide acceptance that diabetes increases severity and mortality from COVID-19.(44)(45) On the other hand, few published researches highlighted the risk of infection of SARS-CoV-2 among diabetics. (46) (47) Although there are some hints of increased susceptibility to infection among diabetics the findings are inconsistent with some researches pointing to a similar prevalence of diabetes in COVID-19 patients to that in the overall population suggesting no relation of diabetes to susceptibility of the infection.(48)(49) Hypertension was another non-communicable disease linked to severity and

fatality of COVID-19 but its relation to the infection risk is much lagging.(50) One limitation of this study is that it depended on self-reporting of hypertension and diabetes.

Younger age group (less than 18 years) expressed the least PCR positivity rate and the least seroprevalence rate (3% and 21% respectively). This observed difference between the different age groups was not statistically significant in the positivity rate but in the seroprevalence analysis. These findings of seroprevalence rate are in line with other researches.(51)(28)(52)(53)

The peak of the first wave in Egypt was in June, 2020 which corresponded well with the highest PCR and positive antibodies detection in the study sample. The seroprevalence rate showed decline in subsequent months which is aligned with other studies. Röltgen et al.showed that outpatient and asymptomatic individuals' SARS-CoV-2 antibodies, including IgG, progressively decreased during observation up to five months post-infection.(54)Findings from some researches proposed a weaker immune response to SARS-CoV-2 infection in asymptomatic individuals and the antibodies level starts to decrease within 2–3 months after infection. (55)(56)Wang et al. also concluded that the antibody level was highest during day 31-40 since onset, and then decreased slightly.(57)

Study limitations: This study was carried out for patients attending Ain Shams University seeking Hospital services, which makes the sample not fully representative of Cairo population. The laboratory tests were not done for all patients for sampling problems, unavailability of certain kits

or laboratory errors. Some data as hypertension and diabetes was based on participant's self-reporting.

Conclusion:

A total of 4313 subjects were enrolled in our study, SARS-Cov-2 PCR was 3.84 % and SARS-CoV-2 antibody seroprevalence was 29.82%. Males had higher PCR detection rate in contrast to females who showed higher seroprevalence rate of anti-SARS-CoV-2 antibodies, younger age group (less than 18 years) expressed the least PCR positivity rate and the least seroprevalence rate. Thus, expanding testing of persons without symptoms should be adopted to reduce silent spread of SARS-CoV-2 in the health care facilities. Our results reinforce the need for continued public health preventive measures, including the use of face masks and social distancing, to limit the spread of SARS-CoV-2.

Authors contributions

- **Samia A Girgis**: Conceptualized and visualized the study, planned and designed the study, planned the study workflow, supervised the study, coordinated and monitored implementation and critically reviewed the manuscript.
- **Hala M Hafez, Hoda Ezz Elarab** and **Basma Sherif:** Laboratory analysis of samples.
- **Moshira H Sabry** and **Iman Afifi**: Supervision and Coordination of implementation, Data acquisition.

- **Fatma Elzahraa Hassan, Amira Reda, Shaimaa Elsayed, Asmaa Mahmoud, Petra Habeb:** Participated in monitoring implementation.
- **Ihab S Habil:** Led data management, conducted statistical data analysis, prepared the tables and figures, interpreted the data, wrote the first draft of the manuscript.
- **Rasha S Hussein:** Data management and analysis
- **Isis M Mossad:** Data management, Drafting the article
- **Ossama Mansour:** Overseeing administrative approvals for the study, Field preparation for the outbreak investigation, Critical revision of the article
- **Ashraf Omar:** Overseeing administrative approvals for the study, Critical revision of the article and approval of the submitted version
- **Ayman M Saleh:** Overseeing administrative approvals for the study, Critical revision of the article
- **Mahmoud El-Meteini:** Overseeing administrative approvals for the study, Critical revision of the article and final approval of the submitted version.

***Potential conflicts of interest*:** All authors report no conflicts of interest relevant to this article.

Acknowledgment: We are thankful to administrative officials at Ain Shams university hospitals for facilitating the conduction of this study. We thank the infection control unit for assistance with data collection also the Laboratory team for testing and screening.

Funding: This study was part of regular policy and procedure of Ain Shams University Hospital

References:

1. Coronavirus Update (Live): 95,076,180 Cases and 2,033,205 Deaths from COVID-19 Virus Pandemic - Worldometer [Internet]. [cited 2021 Jan 17]. Available from: https://www.worldometers.info/coronavirus/

2. Egypt Cares [Internet]. [cited 2021 Jan 17]. Available from: https://www.care.gov.eg/EgyptCare/index.aspx/

3. Ministry of Health and Population Egypt [Internet]. 2021 [cited 2021 Jan 17]. Available from: https://www.who.int/goe/policies/countries/egy_support.pdf?ua=1

4. Roser M, Ritchie H, Ortiz-Ospina E, Hasell J. Coronavirus Pandemic (COVID-19). Our World Data [Internet]. 2020 May 26 [cited 2021 Jan 17]; Available from: https://ourworldindata.org/coronavirus

5. Oran DP, Topol EJ. Prevalence of Asymptomatic SARS-CoV-2 Infection : A Narrative Review [Internet]. Vol. 173, Annals of internal medicine. NLM (Medline); 2020 [cited

2020 Dec 27]. p. 362–7. Available from:
https://www.acpjournals.org/doi/abs/10.7326/M20-3012

6. Arons MM, Hatfield KM, Reddy SC, Kimball A, James A, Jacobs JR, et al. Presymptomatic SARS-CoV-2 Infections and Transmission in a Skilled Nursing Facility. N Engl J Med [Internet]. 2020 May 28 [cited 2021 Jan 17];382(22):2081–90. Available from: https://www.nejm.org/doi/full/10.1056/NEJMoa2008457

7. Hijnen D, GeurtsvanKessel C, Marzano A V., Marzano A V., Eyerich K, Gim nez-Arnau AM, et al. SARS-CoV-2 transmission from presymptomatic meeting attendee, Germany. Emerg Infect Dis [Internet]. 2020 Aug 1 [cited 2021 Jan 17];26(8):1935–7. Available from: https://doi.org/10.1093/aje/kwr113

8. Passarelli VC, Faico-Filho K, Moreira LVL, Luna L, Conte DD, Camargo C, et al. Asymptomatic SARS-CoV-2 infections in hospitalized patients. Infection Control and Hospital Epidemiology. Cambridge University Press; 2020.

9. Flaxman S, Mishra S, Gandy A, Unwin JT, Coupland H, Mellan TA, et al. Report 13: Estimating the number of infections and the impact of non-pharmaceutical interventions on COVID-19 in 11 European countries. [cited 2021 Jan 4]; Available from: https://doi.org/10.25561/77731.

10. Li R, Pei S, Chen B, Song Y, Zhang T, Yang W, et al. Substantial undocumented infection facilitates the rapid dissemination of novel coronavirus (SARS-CoV-2). Science (80-) [Internet]. 2020 May 1 [cited 2021 Jan 4];368(6490):489–93. Available from: https://pubmed.ncbi.nlm.nih.gov/32179701/

11. Hortaçsu A, Liu J, Schwieg T. Estimating the fraction of unreported infections in epidemics with a known epicenter: An application to COVID-19. J Econom [Internet]. 2020 Jan 1 [cited 2021 Jan 4];220(1):106. Available from: /pmc/articles/PMC7476454/?report=abstract

12. Neilan AM, Losina E, Bangs AC, Flanagan C, Panella C, Eskibozkurt GE, et al. Clinical impact, costs, and cost-effectiveness of expanded SARS-CoV-2 testing in Massachusetts [Internet]. medRxiv. medRxiv; 2020 [cited 2021 Jan 17]. Available from: https://pubmed.ncbi.nlm.nih.gov/32743604/

13. Coronavirus (COVID-19) Update: Serological Tests | FDA [Internet]. [cited 2021 Jan 4]. Available from: https://www.fda.gov/news-events/press-announcements/coronavirus-covid-19-update-serological-tests

14. Arevalo-Rodriguez I, Buitrago-Garcia D, Simancas-Racines D, Zambrano-Achig P, Del Campo R, Ciapponi A, et al. False-negative results of initial RT-PCR assays for COVID-19: A systematic review [Internet]. medRxiv. medRxiv; 2020 [cited 2021 Feb 7]. p.

2020.04.16.20066787. Available from: https://doi.org/10.1101/2020.04.16.20066787

15. Public health surveillance for COVID-19: interim guidance [Internet]. [cited 2021 Jan 18]. Available from: https://www.who.int/publications/i/item/who-2019-nCoV-surveillanceguidance-2020.8

16. Tuite AR, Ng V, Rees E, Fisman D, Wilder-Smith A, Khan K, et al. Estimation of the COVID-19 burden in Egypt through exported case detection [Internet]. Vol. 20, The Lancet Infectious Diseases. Lancet Publishing Group; 2020 [cited 2021 Jan 18]. p. 894. Available from: https://doi.org/10.1016

17. Centre for Disease Prevention E. Methodology for estimating point prevalence of SARS-CoV-2 infection by pooled RT-PCR testing. 2020;(May). Available from: https://www.ecdc.europa.eu/sites/default/files/documents/Methodology-estimating-point-prevalence -SARS-CoV-2-infection-pooled-RT-PCR-testing.pdf

18. Lai CC, Wang CY, Ko WC, Hsueh PR. In vitro diagnostics of coronavirus disease 2019: Technologies and application. Journal of Microbiology, Immunology and Infection. Elsevier Ltd; 2020.

19. Eckerle I, Meyer B. SARS-CoV-2 seroprevalence in COVID-19 hotspots [Internet]. Vol. 396, The Lancet. Lancet Publishing Group; 2020. p. 514–5. Available from:

https://doi.org/10.1038/s41591-020-0965-6.

20. Ko JH, Joo EJ, Kim SH, Kim YJ, Huh K, Cho SY, et al. Clinical application of rapid diagnostic test kit for SARS-CoV-2 antibodies into the field of patient care. J Microbiol Immunol Infect. 2020 Jul 10; Available from: https://doi.org/10.1016/j.jmii.2020.07.003

21. Bajema KL, Wiegand RE, Cuffe K, Patel S V., Iachan R, Lim T, et al. Estimated SARS-CoV-2 Seroprevalence in the US as of September 2020. JAMA Intern Med. 2020;1–11. Available from: https://doi.org/10.1001/jamainternmed.2020.7976

22. Jespersen S, Mikkelsen S, Greve T, et al. SARS-CoV-2 seroprevalence survey among 17,971 healthcare and administrative personnel at hospitals, pre-hospital services, and specialist practitioners in the Central Denmark Region. Clinical Infectious Diseases : an Official Publication of the Infectious Diseases Society of America. 2020. DOI: 10.1093/cid/ciaa1471.

23. Stefanelli P, Bella A, Fedele G, Pancheri S, Leone P, Vacca P, et al. Prevalence of SARS-CoV-2 IgG antibodies in an area of northeastern Italy with a high incidence of COVID-19 cases: a population-based study. Clin Microbiol Infect. 2020. Available from: https://doi.org/10.1016/j.cmi.2020.11.013

24. Pollán M, Pérez-Gómez B, Pastor-Barriuso R, Oteo J, Hernán MA, Pérez-Olmeda M, et al. Prevalence of SARS-CoV-2 in Spain (ENE-COVID): a nationwide, population-based

seroepidemiological study. Lancet. 2020;396(10250):535–44. Available from: https://doi.org/10.1016/

25. Poustchi H, Darvishian M, Mohammadi Z, Shayanrad A, Delavari A, Bahadorimonfared A, et al. SARS-CoV-2 antibody seroprevalence in the general population and high-risk occupational groups across 18 cities in Iran: a population-based cross-sectional study. Lancet Infect Dis. 2020.Available from: https://linkinghub.elsevier.com/retrieve/pii/S1473309920308586

26. Stringhini S, Wisniak A, Piumatti G, Azman AS, Lauer SA, Baysson H, et al. Seroprevalence of anti-SARS-CoV-2 IgG antibodies in Geneva, Switzerland (SEROCoV-POP): a population-based study. Lancet. 2020;396:313–9. Available from: https://github.com/HopkinsIDD/

27. Xu X, Sun J, Nie S, Li H, Kong Y, Liang M, et al. Seroprevalence of immunoglobulin M and G antibodies against SARS-CoV-2 in China. Nat Med. 2020;26(8):1193–5. Available from: https://pubmed.ncbi.nlm.nih.gov/32504052/

28. Havers FP, Reed C, Lim T, Montgomery JM, Klena JD, Hall AJ, et al. Seroprevalence of Antibodies to SARS-CoV-2 in 10 Sites in the United States, March 23-May 12, 2020. JAMA Intern Med. 2020;180(12):1576–86. Available from: https://jamanetwork.com/

29. Biggs HM, Harris JB, Breakwell L, Dahlgren FS, Abedi GR, Szablewski CM, et al. Estimated Community Seroprevalence of SARS-CoV-2 Antibodies — Two Georgia Counties, April 28–May 3, 2020. MMWR Morb Mortal Wkly Rep. 2020;69(29):965–70. Available from: https://pubmed.ncbi.nlm.nih.gov/32701941/

30. Chen N, Zhou M, Dong X, Qu J, Gong F, Han Y, et al. Epidemiological and clinical characteristics of 99 cases of 2019 novel coronavirus pneumonia in Wuhan, China: a descriptive study. Lancet. 2020;395(10223):507–13. Available from: https://pubmed.ncbi.nlm.nih.gov/32007143/

31. Guan W, Ni Z, Hu Y, Liang W, Ou C, He J, et al. Clinical Characteristics of Coronavirus Disease 2019 in China. N Engl J Med. 2020;382(18):1708–20. Available from: https://www.nejm.org/doi/full/10.1056/NEJMoa2002032

32. Marcello RK, Dolle J, Grami S, Adule R, Li Z, Tatem K, et al. Characteristics and outcomes of COVID-19 patients in New York City’s public hospital system [Internet]. medRxiv. medRxiv; 2020. p. 2020.05.29.20086645. Available from: https://doi.org/10.1101/2020.05.29.20086645

33. Klein SL, Dhakal S, Ursin RL, Deshpande S, Sandberg K, Mauvais-Jarvis F. Biological sex impacts COVID-19 outcomes. Coyne CB, editor. PLOS Pathog.2020;16(6):e1008570. Available from: https://dx.plos.org/10.1371/journal.ppat.1008570

34. Mazumder A, Arora M, Bharadiya V, Berry P, Agarwal M, Behera P, et al. SARS-CoV-2 epidemic in India: epidemiological features and in silico analysis of the effect of interventions. F1000Research. 2020;9:315. Available from: https://pubmed.ncbi.nlm.nih.gov/32528664/

35. Nikpouraghdam M, Jalali Farahani A, Alishiri GH, Heydari S, Ebrahimnia M, Samadinia H, et al. Epidemiological characteristics of coronavirus disease 2019 (COVID-19) patients in IRAN: A single center study. J Clin Virol. 2020;127:104378. Available from: /pmc/articles/PMC7172806/?report=abstract

36. Wang D, Yin Y, Hu C, Liu X, Zhang X, Zhou S, et al. Clinical course and outcome of 107 patients infected with the novel coronavirus, SARS-CoV-2, discharged from two hospitals in Wuhan, China. Crit Care. 2020;24(1):188. Available from: https://ccforum.biomedcentral.com/articles/10.1186/s13054-020-02895-6

37. The Sex, Gender and COVID-19 Project | Global Health 50/50. [cited 2021 Jan 17]. Available from: https://globalhealth5050.org/the-sex-gender-and-covid-19-project/

38. O'Brien J, Du KY, Peng C. Incidence, clinical features, and outcomes of COVID-19 in Canada: impact of sex and age. J Ovarian Res. 2020;13(1):137. Available from: https://ovarianresearch.biomedcentral.com/articles/10.1186/s13048-020-00734-4

39. Jin JM, Bai P, He W, Wu F, Liu XF, Han DM, et al. Gender Differences in Patients With COVID-19: Focus on Severity and Mortality. Front Public Heal. 2020;8:152. doi: 10.3389/fpubh.2020.00152. PMID: 32411652; PMCID: PMC7201103..

40. Falahi S, Kenarkoohi A. Sex and gender differences in the outcome of patients with COVID-19. J Med Virol. 2021;93(1):151–2. Available from: https://doi.org/10.1002/jmv.26243

41. Fischer J, Jung N, Robinson N, Lehmann C. Sex differences in immune responses to infectious diseases. Vol. 43, Infection. Urban und Vogel GmbH; 2015. p. 399–403. Available from: https://pubmed.ncbi.nlm.nih.gov/25956991/

42. Gebhard C, Regitz-Zagrosek V, Neuhauser HK, Morgan R, Klein SL. Impact of sex and gender on COVID-19 outcomes in Europe . Vol. 11, Biology of Sex Differences. BioMed Central Ltd.; 2020. Available from: /pmc/articles/PMC7247289/?report=abstract

43. Chanana N, Palmo T, Sharma K, Kumar R, Graham BB, Pasha Q. Sex-derived attributes contributing to SARS-CoV-2 mortality. Vol. 319, American Journal of Physiology - Endocrinology and Metabolism. American Physiological Society; 2020. p. E562–7. Available from: http://www.ajpendo.org

44. Fu L, Wang B, Yuan T, Chen X, Ao Y, Fitzpatrick T, et al. Clinical characteristics of

coronavirus disease 2019 (COVID-19) in China: A systematic review and meta-analysis. J Infect. 2020;80(6):656–65. Available from: https://doi.org/10.1016/j.jinf.2020.03.041

45. Yang X, Yu Y, Xu J, Shu H, Xia J, Liu H, et al. Clinical course and outcomes of critically ill patients with SARS-CoV-2 pneumonia in Wuhan, China: a single-centered, retrospective, observational study. Lancet Respir Med. 2020;8(5):475–81. Available from: https://doi.org/10.1016/

46. Erener S. Diabetes, infection risk and COVID-19. Vol. 39, Molecular Metabolism. Elsevier GmbH; 2020. p. 101044. Available from: /pmc/articles/PMC7308743/?report=abstract

47. Kumar A, Gupta R, Ghosh A, Misra A. Diabetes & Metabolic Syndrome : Clinical Research & Reviews Diabetes in COVID-19 : Prevalence , pathophysiology , prognosis and practical considerations. Diabetes Metab Syndr Clin Res Rev. 2020. Available from: https://doi.org/10.1016/j.dsx.2020.04.004

48. Fadini GP, Morieri · M L, Longato · E, Avogaro · A. Prevalence and impact of diabetes among people infected with SARS-CoV-2. J Endocrinol Invest. 2020;43:867–9. Available from: https://doi.org/10.1007/s40618-020-01236-2

49. Ugwueze C V., Ezeokpo BC, Nnolim BI, Agim EA, Anikpo NC, Onyekachi KE. COVID-

19 and Diabetes Mellitus: The Link and Clinical Implications. Dubai Diabetes Endocrinol J. 2020;26(2):69–77. Available from: https://doi.org/10.1159/000511354

50. Schiffrin EL, Flack JM, Ito S, Muntner P, Webb RC. Hypertension and COVID-19. Am J Hypertens. 2020;33(5):373–4. Available from: https://doi.org/10.1093/ajh/hpaa057

51. Sutton M, Cieslak P, Linder M. Morbidity and Mortality Weekly Report Notes from the Field Seroprevalence Estimates of SARS-CoV-2 Infection in Convenience Sample-Oregon. Available from: https://doi.org/10.1128/JCM.00941-20

52. Pollán M, Pérez-Gómez B, Pastor-Barriuso R, Oteo J, Hernán MA, Pérez-Olmeda M, et al. Prevalence of SARS-CoV-2 in Spain (ENE-COVID): a nationwide, population-based seroepidemiological study. Lancet. 2020;396(10250):535–44. Available from: https://doi.org/10.1016/S0140-6736(20)31483-5

53. Stringhini S, Wisniak A, Piumatti G, Azman AS, Lauer SA, Baysson H, et al. Seroprevalence of anti-SARS-CoV-2 IgG antibodies in Geneva, Switzerland (SEROCoV-POP): a population-based study. Lancet. 2020;396(10247):313–9. Available from:

https://dx.doi.org/10.1016%2FS0140-6736(20)31304-0

54. Röltgen K, Powell AE, Wirz OF, Stevens BA, Hogan CA, Najeeb J, et al. Defining the features and duration of antibody responses to SARS-CoV-2 infection associated with

disease severity and outcome. Sci Immunol. 2020;5(54). Available from: http://immunology.sciencemag.org/

55. Ibarrondo FJ, Fulcher JA, Goodman-Meza D, Elliott J, Hofmann C, Hausner MA, et al. Rapid Decay of Anti–SARS-CoV-2 Antibodies in Persons with Mild Covid-19. N Engl J Med. 2020;383(11):1085–7. Available from: https://www.nejm.org/doi/full/10.1056/nejmc2025179

56. Long QX, Tang XJ, Shi QL, Li Q, Deng HJ, Yuan J, et al. Clinical and immunological assessment of asymptomatic SARS-CoV-2 infections. Nat Med. 2020;26(8):1200–4. Available from:https://doi.org/10.1038/s41591-020-0965-6

57. Wang X, Guo X, Prof A, Xin Q, Pan Y, Li J, et al. Neutralizing Antibodies Responses to SARS-CoV-2 in COVID-19 Inpatients and Convalescent Patients. medRxiv. 2020;2020.04.15.20065623. Available from: https://doi.org/10.1101/2020.04.15.20065623

Tables

(Table 1) Characteristics of the study group

	No. (%)
**Total no**	4313
**Age (years)**	
**<18**	928 (21.5)
**18-**	1356 (31.4)
**40-**	1214 (28.1)
**≥60**	815 (18.9)
**Gender**	

**Male**	1885 (43.7)
**Females**	2428 (56.3)
**Hospital**	
**1. Obstetrics and gynecology**	703 (16.3)
**2. Oncology**	49 (1.1)
**3. Surgery**	1463 (33.9)
**4. Pediatrics**	443 (10.3)
**5. Internal medicine**	1421 (32.9)
**6. Cardiothoracic**	234 (5.4)
**Symptoms**	
**1. No COVID-19 related symptoms**	3939 (91.3)
**2. fever**	262 (6.1)
**3. cough**	165 (3.8)
**4. diarrhea**	85 (2.0)
**5. sore throat**	106 (2.5)
**6. vascular event**	44 (1.0)
**Morbidities**	

**1. DM (N=3659)**	298 (8.1)
**2. HTN (N= 3659)**	352 (9.6)
**No. of PCR done**	4008 (92.9)
**No. of AB assay done**	2951 (68.4)

Table 2 Results of SARS-CoV-2 screening by PCR and total antibody

	No. (unadjusted %, 95% CI)	Adjusted * % (95% CI)
**Positive PCR in total group (N=4008)**	154 (3.84, 3.29-4.48)	Scenario1 (sensitivity 71%)<br>5.28 (4.47- 6.18)<br>Scenario 2 (sensitivity 95%)<br>3.94 (3.34 -4.62)
**Positive Antibody among negative PCR group (N=2927**)**	877(29.96, 28.31-31.66)	29.82 (28.16-31.51)
**Negative PCR and Negative AB(N=2927*)**	1927(65.84, 64,1-67.53)	

**Positive PCR and Negative AB (N=2927*)**	55 (1.88, 1.45-2.44)
**Positive PCR and positive AB (N=2927*)**	68 (2.32, 1.84-2.94)

* Adjustment for sensitivity and specificity of the test

**The total number of subjects with both tests (PCR and total AB) determined

Table 3: Epidemiological profile of SARS-C-V-2 PCR positive and antibody seropositive subjects

	Total no.	PCR +ve No. (%)	Adjusted OR (95% CI)	Total no.	AB +ve* No. (%)	Adjusted OR (95% CI)
			All age groups			
**Age (years)**						
< 18	841	25 (3.0)	1	642	135 (21.0)	1
18-	1268	43(3.4)	1.067(0.66 -1.73)	923	361 (39.1)	2.19 (1.73-2.77)
40-	1136	54(4.8)	1.39 (0.87 -2.26)	768	273 (35.5)	1.94 (1.52-2.47)
≥60	763	32(4.2)	1.55 (0.933- 2.62)	471	108 (22.9)	1.05 (0.79-1.40)

**Gender**						
Male	1732	76 (4.4)	1.45(1.06 -1.98)	1258	313 (24.9)	0.63(0.53- 0.75)
Female	2276	78 (3.4)	1	1546	564 (36.5)	1
Adult Group (>18 years)						
**Age (years)**						
18-	1268	43(3.4)	1	923	361 (39.1)	1
40-	1136	54(4.8)	1.42 (0.89 -2.27)	768	273 (35.5)	0.93 (0.75-1.16)
≥60	763	32(4.2)	1.2 (0.73- 2.01)	471	108 (22.9)	0.48(0.37-0.77)
**Gender**						
Male	1255	68 (5.4)	1.63 (1.09-2.43)	878	241(27.4)	0.63 (0.52-0.77)
Female	1912	61 (3.2)	1	1284	501 (39)	1
**DM**						
Negative	2470	93 (3.8)	1	1794	597 (33.3)	1
Positive	265	9 (3.4)	1.19(0.56 -2.52)	142	44 (31.0)	1.03 (0.7 -1.56)
**HTN**						
Negative	2398	95 (4.0)	1	1732	579 (33.4)	1
Positive	337	7(2.1)	0.47 (0.20 - 1.08)	204	62 (30.4)	0.92 (0.65 -1.31)

* The percentage is calculated among the PCR-ve group

Figure

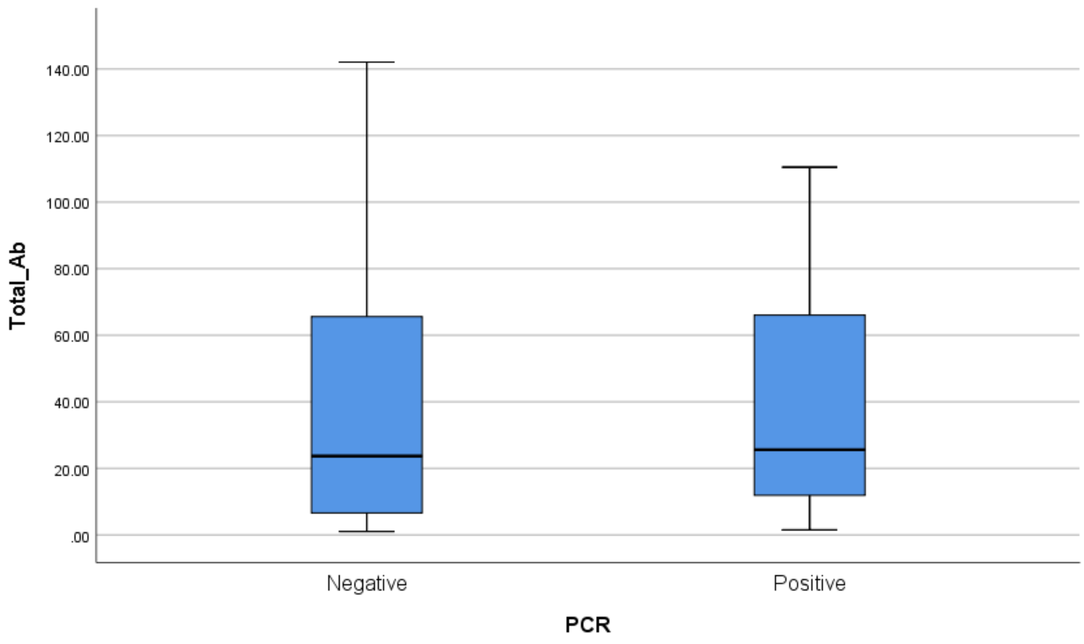

Figure 1 Box plot of antibody level in the positively tested antibody group
