# Supplementary material for: SARS-CoV-2 PCR positivity rate and seroprevalence of related antibodies among a sample of patients in Cairo: Pre-wave 2 results of a screening program in a university hospital
Source: PLoS One. 2021 Jul 15;16(7):e0254581. doi: 10.1371/journal.pone.0254581 (PMC8282003; doi:10.1371/journal.pone.0254581)
Supplement: Attachment — Submitted filename: Response to reviwers.docx [file pone.0254581.s005.docx]

SARS-CoV-2 PCR positivity rate and seroprevalence of related antibodies among a sample of patients in Cairo: Pre-wave 2 results of a screening program in a university hospital

| **Editor comments** | |
| --- | --- |
| Please include the following items when submitting your revised manuscript: | |
| A rebuttal letter that responds to each point raised by the academic editor and reviewer(s). You should upload this letter as a separate file labeled 'Response to Reviewers'. | On resubmission  done |
| A marked-up copy of your manuscript that highlights changes made to the original version. You should upload this as a separate file labeled 'Revised Manuscript with Track Changes | On resubmission  done |
| An unmarked version of your revised paper without tracked changes. You should upload this as a separate file labeled 'Manuscript | On resubmission  done |
| The introduction should be updated to appropriately reflect the current number of infections in Egypt. | Done |
| There are a number of awkward phrases and grammatical / spelling mistakes.  The manuscript should be reviewed carefully by a native English speaker and/or a professional editing service. | The manuscript was reviewed by a professional editing service. |
| The methods should include an estimate of how many unique individuals are seen in this university hospital setting per year. | Referred to page 4 line 14 .This statement was added to the study setting in the methodology "It a campus including seven hospitals (surgery hospital, internal medicine hospital, pediatrics hospital, obstetrics, and gynecology hospital, emergency hospital , and geriatrics hospital), and multiple specific units and centers as clinical toxicology center, psychiatric center, bone marrow transplant unit, digestive endoscopy unit and clinical research unit. It involves more than 3000 inpatient beds, in addition to ICUs at the different hospitals., and serving about one and a half million patients annually" |
| It is unclear why extracted RNA was kept at -20C.  This almost certainly led to sample degradation prior to RT-PCR testing. | Most of the time RNA was processed immediately after extraction except in few times where the PCR instrument is busy and we had to wait for less than 2 hours to process the samples. Therefore, extracts we stored at the -20 in accordance to manufacturer’s recommendations. |
| Was real-time PCR performed in duplicate for each sample? | In page 8 line 2 we mentioned that samples were processed only once |
| Were RT-PCR negative samples re-tested to confirm the negative finding? | In this study we aimed to screen patients admitted to the hospital for medical and/or surgical purposes. Usually, they were symptom-free and hence there was no need to repeat the testing. Yet, only in suspected cases, with CT findings suggestive of SARS-COV2 infection, re-sampling and re-testing was done. |
| Were symptoms and co-morbid conditions determined by self-report or was fever actually measured?  Were co-morbid conditions determined by patient chart review? | Refereed to page 4 line 6.  Regarding patients' symptoms body temperature was measured for all patients as it’s a part of patient general evaluation that done on admission, other symptoms like sore throat and cough were collected based on patient self-reporting  Regarding co-morbid conditions, it was collected also, based on the patients self-reporting and we mentioned this point as one of our study limitations |
| Figure 1 can be simplified by removing the horizontal lines and by give antibodies levels as 0 to 140 (rather than .00 to 140.00). | Done |
| **Journal requirements comments** | |
| Please ensure that your manuscript meets PLOS ONE's style requirements, including those for file naming. | |
| Please provide additional details regarding participant consent. In the ethics statement in the Methods and online submission information, please ensure that you have specified   (1) whether consent was informed (2) what type you obtained (for instance, written or verbal, and if verbal, how it was documented and witnessed). (3) If your study included minors, state whether you obtained consent from parents or guardians. If the need for consent was waived by the ethics committee, please include this information. | Referred to page 10 line 14  Yes, all participant provided documented written consent, the following statement was added at the ethical considerations section.  " A written informed consent in Arabic was obtained from participants or their guardians in the case of minors (Arabic and English translations of the consent form are attached in the supplementary materials" |
| If you are reporting a retrospective study of medical records or archived samples, please ensure that you have discussed whether all data were fully anonymized before you accessed them and/or whether the IRB or ethics committee waived the requirement for informed consent. If patients provided informed written consent to have data from their medical records used in research, please include this information. | In our study we made active data collection, we didn't used archived data |
| Please amend your current ethics statement to include the full name of the ethics committee/institutional review board(s) that approved your specific study.  Once you have amended this/these statement(s) in the Methods section of the manuscript, please add the same text to the “Ethics Statement” field of the submission form (via “Edit Submission”). | Refereed to page 10 line 11  The following statement was added to the ethical considerations section " The protocol of the research was approved by the University Ethical Research Committee [ FWA00017585] , the research approval number (FMASU R /2020)" |
| , please provide the following:   - The name of the colleague or the details of the professional service that edited your manuscript - A copy of your manuscript showing your changes by either highlighting them or using track changes (uploaded as a *supporting information* file) - A clean copy of the edited manuscript (uploaded as the new *manuscript* file | On resubmission  done |
| please include additional information regarding the survey or questionnaire used in the study and ensure that you have provided sufficient details that others could replicate the analyses. For instance, if you developed a questionnaire as part of this study and it is not under a copyright more restrictive than CC-BY, please include a copy, in both the original language and English, as Supporting Information. Moreover, please include more details on how the questionnaire was pre-tested, and whether it was validated. | Done |
| Please ensure that you refer to Figure 1 in your text as, if accepted, production will need this reference to link the reader to the figure. | Done |
| We note you have included a table to which you do not refer in the text of your manuscript. Please ensure that you refer to Table 1, 2 and 3 in your text; if accepted, production will need this reference to link the reader to the Table. | Done |
| We noticed you have some minor occurrence of overlapping text with the following previous publication(s), which needs to be addressed: - https://ovarianresearch.biomedcentral.com/articles/10.1186/s13048-020-00734-4 - https://www.clinicalmicrobiologyandinfection.com/article/S1198-743X(20)30706-0/fulltext In your revision ensure you cite all your sources (including your own works), and quote or rephrase any duplicated text outside the methods section. Further consideration is dependent on these concerns being addressed | Done |
| **Reviewer 1** | |
| Do you have anti- SARS-CoV-2 IgM antibodies especially in PCR positive samples? | In SARS-CoV-2 infections, IgM and IgG antibodies can arise nearly simultaneously in serum within 2 to 3 weeks after illness onset. Thus, detection of IgM without IgG is uncommon (CDC, 2020). That is why we used the ROCHE chemiluminescence immunoassay (Elecsys anti-SARS-COV2 total) which is qualitative assay that is used to detect the total antibodies (including IgG) against SARS-COV2. |
| The discrepancy between the PCR positive cases and antibodies negative needs to be addressed and explained since about half of the PCR positive patients are antibodies negative. Is it because of early testing and if so what about the anti- SARS-CoV-2 IgM antibodies, or because of the false positive PCR or the false negative antibodies | The discrepancy between the PCR positive cases and antibodies negative may be explained by:   1) Antibodies usually start to appear 7 to 10 days post-infection and even become detectable during the third week as recommended by the CDC. By that time the patient infectiousness start to decline. The antibodies determine whether the patient has been previously exposed to infection but cannot be used to assess the current infection status. 2) `33% of COVID 19 patients were found to have no detectable antibodies. That is why the CDC recommended not to use the antibody test neither in the diagnosis of COVID-19 cases nor in the determination of immunity.   Refer to CDC Interim Guidelines for COVID-19 Antibody Testing |
| Do you have samples to follow some of the PCR positive antibodies negative to evaluate if they will be seroconverted | This was beyond the scope of the study. |
| Cross-reactive antibodies to other Corona viruses should be considered especially for PCR negative antibodies positive samples and may be needed to be discussed or confirmed with other serological kits | The analytical specificity of the Elecsys anti-SARS COV2 assay is 99.5% as determined by the manufacturer (Elecsys anti-SARS COV2 assay package insert). A total of 792 potentially cross-reacting samples were tested and no-cross reactivity has been reported with the common cold panel (number of tested samples = 40) nor the coronavirus panel (number of tested samples = 40). |
| **Reviewer 2** | |
| **Introduction** COV-2 over the entire population is likely much higher, and accordingly the mortality rate should be fundamentally lower.  What are these numbers? Agencies sited?  The total number of screening tests done in Egypt has been substantially lower than that done in other countries  The number at writing the manuscript should be included | Numbers of daily PCR test that conducted daily at the time of writing the manuscript was added " Page 2 line 14" |
| as duration antibodies in patients after infection  this statement is incomplete | The statement was corrected |
| Gynecology and Obstetrics, Oncology, Pediatrics, Psychiatry, Internal Medicine, surgical, cardiothoracic and Geriatrics  Are these different hospitals or departments within the same hospital | Yes, these are different hospitals.  Ain Shams university hospitals include 7 hospitals (surgery hospital, internal medicine hospital, pediatrics hospital, obstetrics, and gynecology hospital, emergency hospital , and geriatrics hospital), and multiple specific units and centers as clinical toxicology center, psychiatric center, bone marrow transplant unit, digestive endoscopy unit and clinical research unit. It involves more than 3000 inpatient beds, in addition to ICUs at the different hospitals. |
| **Methodology:**  Nucleic acid extraction: Viral RNA was extracted using the Viasure RNA/DNA extraction kit (CerTest Biotec, Spain) as follows: − Each sample was mixed well by gentle vortexing and a volume of 300ul was transferred into a deep-well plate. − A total volume of 314ul lysing solution containing 300ul of lysis buffer, 10ul proteinase K solution and 4ul of carrier RNA was added into each sample well. − Sample plate was then loaded onto the Chemagic 360 automatic extractor (Perkin Elmer, Germany). Viral RNA was then automatically extracted by binding to the surface of magnetic beads. Impurities such as salts, metabolites, and soluble macromolecular cellular components were efficiently removed by a series of quick washing steps. − Unless immediately processed, extracted RNA was kept at -20°C.  **Comment : follow manufacturers protocol** | Done |
| Ethical considerations:  The protocol of the research was approved by the University Ethical Research Committee  Comment: Insert the approval number | Done |
| **Results:** The current research enrolled 4313 subjects during the study period. A total of 4008 and 2951 patients had the PCR test and the antibody assay done, respectively. Females constituted 56% of the study sample. Adults and middle age represented around 60% of the sample. The vast majority of patients (91.3%) did not complain of any related COVID-19 symptoms.  Comment : table or figure should be stated | Done |
| Males showed higher risk for getting the COVID-19 infection as detected by positive PCR (OR adjusted for age was 1.45, 95% CI 1.06 -1.98). On analyzing the adult group separately for comorbid conditions, males preserved their risk differential after adjustment for comorbid conditions (Diabetes and hypertension). Age and comorbid conditions did not show any significant relation to PCR positivity rate  **Comments: no tables or figures** | Done |
