# Supplementary material for: SARS-CoV-2 PCR positivity rate and seroprevalence of related antibodies among a sample of patients in Cairo: Pre-wave 2 results of a screening program in a university hospital
Source: PLoS One. 2021 Jul 15;16(7):e0254581. doi: 10.1371/journal.pone.0254581 (PMC8282003; doi:10.1371/journal.pone.0254581)
Supplement: Peer review history

# Peer review: Decision Letter 0

**DOI:** 10.1371/journal.pone.0254581.r001

**Authors:** Jason T. Blackard

14 Apr 2021

PONE-D-21-06534

SARS-CoV-2 PCR positivity rate and seroprevalence of related antibodies among a sample of patients in Cairo: Pre-wave 2 results of a screening program in a university hospital

PLOS ONE

Dear Dr. Mossad,

Thank you for submitting your manuscript to PLOS ONE. After careful consideration, we feel that it has merit but does not fully meet PLOS ONE’s publication criteria as it currently stands. Therefore, we invite you to submit a revised version of the manuscript that addresses the points raised during the review process.

Please submit your revised manuscript by May 28 2021 11:59PM. If you will need more time than this to complete your revisions, please reply to this message or contact the journal office at plosone@plos.org. When you're ready to submit your revision, log on to https://www.editorialmanager.com/pone/ and select the 'Submissions Needing Revision' folder to locate your manuscript file.

Please include the following items when submitting your revised manuscript:

- A rebuttal letter that responds to each point raised by the academic editor and reviewer(s). You should upload this letter as a separate file labeled 'Response to Reviewers'.
- A marked-up copy of your manuscript that highlights changes made to the original version. You should upload this as a separate file labeled 'Revised Manuscript with Track Changes'.
- An unmarked version of your revised paper without tracked changes. You should upload this as a separate file labeled 'Manuscript'.

If you would like to make changes to your financial disclosure, please include your updated statement in your cover letter. Guidelines for resubmitting your figure files are available below the reviewer comments at the end of this letter.

If applicable, we recommend that you deposit your laboratory protocols in protocols.io to enhance the reproducibility of your results. Protocols.io assigns your protocol its own identifier (DOI) so that it can be cited independently in the future. For instructions see: http://journals.plos.org/plosone/s/submission-guidelines#loc-laboratory-protocols. Additionally, PLOS ONE offers an option for publishing peer-reviewed Lab Protocol articles, which describe protocols hosted on protocols.io. Read more information on sharing protocols at https://plos.org/protocols?utm_medium=editorial-email&utm_source=authorletters&utm_campaign=protocols.

We look forward to receiving your revised manuscript.

Kind regards,

Jason T. Blackard, PhD

Academic Editor

PLOS ONE

Additional Editor Comments:

This is a cross-sectional study of SARS-CoV-2 antibody and RNA status in a university hospital setting in Egypt.  

The population size studied is large and had an RNA positivity rate of 3.84%.

The introduction should be updated to appropriately reflect the current number of infections in Egypt.

There are a number of awkward phrases and grammatical / spelling mistakes.  The manuscript should be reviewed carefully by a native English speaker and/or a professional editing service.

The methods should include an estimate of how many unique individuals are seen in this university hospital setting per year.  

It is unclear why extracted RNA was kept at -20C.  This almost certainly led to sample degradation prior to RT-PCR testing.

Was real-time PCR performed in duplicate for each sample?

Were RT-PCR negative samples re-tested to confirm the negative finding?

Were symptoms and co-morbid conditions determined by self-report or was fever actually measured?  Were co-morbid conditions determined by patient chart review?

Figure 1 can be simplified by removing the horizontal lines and by give antibodies levels as 0 to 140 (rather than .00 to 140.00).

Journal Requirements:

When submitting your revision, we need you to address these additional requirements.

1. Please ensure that your manuscript meets PLOS ONE's style requirements, including those for file naming. The PLOS ONE style templates can be found at

https://journals.plos.org/plosone/s/file?id=wjVg/PLOSOne_formatting_sample_main_body.pdf and

https://journals.plos.org/plosone/s/file?id=ba62/PLOSOne_formatting_sample_title_authors_affiliations.pdf

2. Please provide additional details regarding participant consent. In the ethics statement in the Methods and online submission information, please ensure that you have specified (1) whether consent was informed and (2) what type you obtained (for instance, written or verbal, and if verbal, how it was documented and witnessed). If your study included minors, state whether you obtained consent from parents or guardians. If the need for consent was waived by the ethics committee, please include this information.

If you are reporting a retrospective study of medical records or archived samples, please ensure that you have discussed whether all data were fully anonymized before you accessed them and/or whether the IRB or ethics committee waived the requirement for informed consent. If patients provided informed written consent to have data from their medical records used in research, please include this information.

3. Thank you for including your ethics statement:  "The research was approved by the University Ethical Research Committee.

FW A00017585 assurance number, Positive cases were reported to the Ministry of Health and Population (MOHP). The guidelines of isolation and treatment protocol of MOHP were followed."

Please amend your current ethics statement to include the full name of the ethics committee/institutional review board(s) that approved your specific study.

Once you have amended this/these statement(s) in the Methods section of the manuscript, please add the same text to the “Ethics Statement” field of the submission form (via “Edit Submission”).

For additional information about PLOS ONE ethical requirements for human subjects research, please refer to http://journals.plos.org/plosone/s/submission-guidelines#loc-human-subjects-research. 

4. We suggest you thoroughly copyedit your manuscript for language usage, spelling, and grammar. If you do not know anyone who can help you do this, you may wish to consider employing a professional scientific editing service.  

Whilst you may use any professional scientific editing service of your choice, PLOS has partnered with both American Journal Experts (AJE) and Editage to provide discounted services to PLOS authors. Both organizations have experience helping authors meet PLOS guidelines and can provide language editing, translation, manuscript formatting, and figure formatting to ensure your manuscript meets our submission guidelines. To take advantage of our partnership with AJE, visit the AJE website (http://learn.aje.com/plos/) for a 15% discount off AJE services. To take advantage of our partnership with Editage, visit the Editage website (www.editage.com) and enter referral code PLOSEDIT for a 15% discount off Editage services.  If the PLOS editorial team finds any language issues in text that either AJE or Editage has edited, the service provider will re-edit the text for free.

Upon resubmission, please provide the following:

- The name of the colleague or the details of the professional service that edited your manuscript
- A copy of your manuscript showing your changes by either highlighting them or using track changes (uploaded as a *supporting information* file)
- A clean copy of the edited manuscript (uploaded as the new *manuscript* file)

5. Please include additional information regarding the survey or questionnaire used in the study and ensure that you have provided sufficient details that others could replicate the analyses. For instance, if you developed a questionnaire as part of this study and it is not under a copyright more restrictive than CC-BY, please include a copy, in both the original language and English, as Supporting Information. Moreover, please include more details on how the questionnaire was pre-tested, and whether it was validated.

6. We note that you have indicated that data from this study are available upon request. PLOS only allows data to be available upon request if there are legal or ethical restrictions on sharing data publicly. For information on unacceptable data access restrictions, please see http://journals.plos.org/plosone/s/data-availability#loc-unacceptable-data-access-restrictions.

In your revised cover letter, please address the following prompts:

a) If there are ethical or legal restrictions on sharing a de-identified data set, please explain them in detail (e.g., data contain potentially identifying or sensitive patient information) and who has imposed them (e.g., an ethics committee). Please also provide contact information for a data access committee, ethics committee, or other institutional body to which data requests may be sent.

b) If there are no restrictions, please upload the minimal anonymized data set necessary to replicate your study findings as either Supporting Information files or to a stable, public repository and provide us with the relevant URLs, DOIs, or accession numbers. Please see http://www.bmj.com/content/340/bmj.c181.long for guidelines on how to de-identify and prepare clinical data for publication. For a list of acceptable repositories, please see http://journals.plos.org/plosone/s/data-availability#loc-recommended-repositories.

We will update your Data Availability statement on your behalf to reflect the information you provide.

7. Thank you for stating the following in the Funding Section of your manuscript:

[This study was part of regular policy and procedure of Ain Shams University Hospital.]

We note that you have provided funding information that is not currently declared in your Funding Statement. However, funding information should not appear in the Acknowledgments section or other areas of your manuscript. We will only publish funding information present in the Funding Statement section of the online submission form.

Please remove any funding-related text from the manuscript and let us know how you would like to update your Funding Statement. Currently, your Funding Statement reads as follows:

 [the authors didn't receive any specific funding for their work.]

Please include your amended statements within your cover letter; we will change the online submission form on your behalf.

8. Please ensure that you refer to Figure 1 in your text as, if accepted, production will need this reference to link the reader to the figure.

9. We note you have included a table to which you do not refer in the text of your manuscript. Please ensure that you refer to Table 1, 2 and 3 in your text; if accepted, production will need this reference to link the reader to the Table.

10. We noticed you have some minor occurrence of overlapping text with the following previous publication(s), which needs to be addressed:

- https://ovarianresearch.biomedcentral.com/articles/10.1186/s13048-020-00734-4

- https://www.clinicalmicrobiologyandinfection.com/article/S1198-743X(20)30706-0/fulltext

In your revision ensure you cite all your sources (including your own works), and quote or rephrase any duplicated text outside the methods section. Further consideration is dependent on these concerns being addressed.

[Note: HTML markup is below. Please do not edit.]

Reviewers' comments:

Reviewer's Responses to Questions

**Comments to the Author**

1. Is the manuscript technically sound, and do the data support the conclusions?

The manuscript must describe a technically sound piece of scientific research with data that supports the conclusions. Experiments must have been conducted rigorously, with appropriate controls, replication, and sample sizes. The conclusions must be drawn appropriately based on the data presented. 

Reviewer #1: Partly

Reviewer #2: Partly

**********

2. Has the statistical analysis been performed appropriately and rigorously? 

Reviewer #1: Yes

Reviewer #2: No

**********

3. Have the authors made all data underlying the findings in their manuscript fully available?

The PLOS Data policy requires authors to make all data underlying the findings described in their manuscript fully available without restriction, with rare exception (please refer to the Data Availability Statement in the manuscript PDF file). The data should be provided as part of the manuscript or its supporting information, or deposited to a public repository. For example, in addition to summary statistics, the data points behind means, medians and variance measures should be available. If there are restrictions on publicly sharing data—e.g. participant privacy or use of data from a third party—those must be specified.

Reviewer #1: Yes

Reviewer #2: No

**********

4. Is the manuscript presented in an intelligible fashion and written in standard English?

PLOS ONE does not copyedit accepted manuscripts, so the language in submitted articles must be clear, correct, and unambiguous. Any typographical or grammatical errors should be corrected at revision, so please note any specific errors here.

Reviewer #1: Yes

Reviewer #2: No

**********

5. Review Comments to the Author

Please use the space provided to explain your answers to the questions above. You may also include additional comments for the author, including concerns about dual publication, research ethics, or publication ethics. (Please upload your review as an attachment if it exceeds 20,000 characters)

Reviewer #1: Girgis et al, evaluated the SARS-CoV-2 PCR positivity rate and seroprevalence of anti-SARS-CoV-2 among 4313 patients in Cairo between May 5th and end of October 2020 (Pre-wave 2) in a university hospital. The study is important and addressed interesting questions about the seroprevalence of SARS-CoV-2 in Egypt. To improve the quality of the manuscript, the following should be addressed:

1. Do you have anti- SARS-CoV-2 IgM antibodies especially in PCR positive samples?

2. The discrepancy between the PCR positive cases and antibodies negative needs to be addressed and explained since about half of the PCR positive patients are antibodies negative. Is it because of early testing and if so what about the anti- SARS-CoV-2 IgM antibodies, or because of the false positive PCR or the false negative antibodies?

3. Do you have samples to follow some of the PCR positive antibodies negative to evaluate if they will be seroconverted?

4. Cross-reactive antibodies to other Corona viruses should be considered especially for PCR negative antibodies positive samples and may be needed to be discussed or confirmed with other serological kits.

Reviewer #2: The manuscript titled 'SARS-CoV-2 PCR positivity rate and seroprevalence of related antibodies among a sample of patients in Cairo: Pre-wave 2 results of a screening program in a university hospital' by Girgis et al. contains some important information which may be beneficial to the scientific and policy makers. However, the manuscript in its current state needs to be revised to meet a certain standard.

Most of the issues raised such as comments and edits have are in track changes in the attachment. Unfortunately, since the authors did not insert line numbers, it was difficult writing out a comprehensive report.

**********

6. PLOS authors have the option to publish the peer review history of their article (what does this mean?). If published, this will include your full peer review and any attached files.

If you choose “no”, your identity will remain anonymous but your review may still be made public.

**Do you want your identity to be public for this peer review?** For information about this choice, including consent withdrawal, please see our Privacy Policy.

Reviewer #1: **Yes: **Mohamed Tarek Mohamed Shata

Reviewer #2: No

[NOTE: If reviewer comments were submitted as an attachment file, they will be attached to this email and accessible via the submission site. Please log into your account, locate the manuscript record, and check for the action link "View Attachments". If this link does not appear, there are no attachment files.]

While revising your submission, please upload your figure files to the Preflight Analysis and Conversion Engine (PACE) digital diagnostic tool, https://pacev2.apexcovantage.com/. PACE helps ensure that figures meet PLOS requirements. To use PACE, you must first register as a user. Registration is free. Then, login and navigate to the UPLOAD tab, where you will find detailed instructions on how to use the tool. If you encounter any issues or have any questions when using PACE, please email PLOS at figures@plos.org. Please note that Supporting Information files do not need this step.

Attachment Submitted filename: PONE-D-21-06534_reviewer_AAS.pdf Click here for additional data file.

# Author comment: Author response to Decision Letter 0

**DOI:** 10.1371/journal.pone.0254581.r002

10 Jun 2021

Editor comments

Please include the following items when submitting your revised manuscript:

*A rebuttal letter that responds to each point raised by the academic editor and reviewer(s). You should upload this letter as a separate file labeled 'Response to Reviewers': Done on resubmission.

*A marked-up copy of your manuscript that highlights changes made to the original version. You should upload this as a separate file labeled 'Revised Manuscript with Track Changes: Done on resubmission.

*An unmarked version of your revised paper without tracked changes. You should upload this as a separate file labeled 'Manuscript: done on resubmission.

*The introduction should be updated to appropriately reflect the current number of infections in Egypt: Done.

*There are a number of awkward phrases and grammatical / spelling mistakes. The manuscript should be reviewed carefully by a native English speaker and/or a professional editing service: The manuscript was reviewed by a professional editing service.

*The methods should include an estimate of how many unique individuals are seen in this university hospital setting per year: Referred to page 4 line 14 .This statement was added to the study setting in the methodology "It a campus including seven hospitals (surgery hospital, internal medicine hospital, pediatrics hospital, obstetrics, and gynecology hospital, emergency hospital , and geriatrics hospital), and multiple specific units and centers as clinical toxicology center, psychiatric center, bone marrow transplant unit, digestive endoscopy unit and clinical research unit. It involves more than 3000 inpatient beds, in addition to ICUs at the different hospitals., and serving about one and a half million patients annually".

*It is unclear why extracted RNA was kept at -20C. This almost certainly led to sample degradation prior to RT-PCR testing: Most of the time RNA was processed immediately after extraction except in few times where the PCR instrument is busy and we had to wait for less than 2 hours to process the samples. Therefore, extracts we stored at the -20 in accordance to manufacturer’s recommendations.

*Was real-time PCR performed in duplicate for each sample? In page 8 line 2 we mentioned that samples were processed only once.

*Were RT-PCR negative samples re-tested to confirm the negative finding? In this study we aimed to screen patients admitted to the hospital for medical and/or surgical purposes. Usually, they were symptom-free and hence there was no need to repeat the testing. Yet, only in suspected cases, with CT findings suggestive of SARS-COV2 infection, re-sampling and re-testing was done.

*Were symptoms and co-morbid conditions determined by self-report or was fever actually measured? Were co-morbid conditions determined by patient chart review? Refereed to page 4 line 6.

 Regarding patients' symptoms body temperature was measured for all patients as it’s a part of patient general evaluation that done on admission, other symptoms like sore throat and cough were collected based on patient self-reporting

Regarding co-morbid conditions, it was collected also, based on the patients self-reporting and we mentioned this point as one of our study limitations.

*Figure 1 can be simplified by removing the horizontal lines and by give antibodies levels as 0 to 140 (rather than .00 to 140.00): Done.

Journal requirements comments:

 Please ensure that your manuscript meets PLOS ONE's style requirements, including those for file naming.

 Please provide additional details regarding participant consent. In the ethics statement in the Methods and online submission information, please ensure that you have specified 

(1) whether consent was informed

(2) what type you obtained (for instance, written or verbal, and if verbal, how it was documented and witnessed).

(3) If your study included minors, state whether you obtained consent from parents or guardians. If the need for consent was waived by the ethics committee, please include this information: Referred to page 10 line 14 

Yes, all participant provided documented written consent, the following statement was added at the ethical considerations section.

" A written informed consent in Arabic was obtained from participants or their guardians in the case of minors (Arabic and English translations of the consent form are attached in the supplementary materials" 

If you are reporting a retrospective study of medical records or archived samples, please ensure that you have discussed whether all data were fully anonymized before you accessed them and/or whether the IRB or ethics committee waived the requirement for informed consent. If patients provided informed written consent to have data from their medical records used in research, please include this information.

 In our study we made active data collection, we didn't used archived data 

*Please amend your current ethics statement to include the full name of the ethics committee/institutional review board(s) that approved your specific study.

Once you have amended this/these statement(s) in the Methods section of the manuscript, please add the same text to the “Ethics Statement” field of the submission form (via “Edit Submission”).: Refereed to page 10 line 11

The following statement was added to the ethical considerations section " The protocol of the research was approved by the University Ethical Research Committee [ FWA00017585] , the research approval number (FMASU R /2020)"

, please provide the following:

• The name of the colleague or the details of the professional service that edited your manuscript

• A copy of your manuscript showing your changes by either highlighting them or using track changes (uploaded as a *supporting information* file)

• A clean copy of the edited manuscript (uploaded as the new *manuscript* file

Done on resubmission.

*please include additional information regarding the survey or questionnaire used in the study and ensure that you have provided sufficient details that others could replicate the analyses. For instance, if you developed a questionnaire as part of this study and it is not under a copyright more restrictive than CC-BY, please include a copy, in both the original language and English, as Supporting Information. Moreover, please include more details on how the questionnaire was pre-tested, and whether it was validated: Done.

*Please ensure that you refer to Figure 1 in your text as, if accepted, production will need this reference to link the reader to the figure: Done.

 *We note you have included a table to which you do not refer in the text of your manuscript. Please ensure that you refer to Table 1, 2 and 3 in your text; if accepted, production will need this reference to link the reader to the Table: Done.

*We noticed you have some minor occurrence of overlapping text with the following previous publication(s), which needs to be addressed:

- https://ovarianresearch.biomedcentral.com/articles/10.1186/s13048-020-00734-4

- https://www.clinicalmicrobiologyandinfection.com/article/S1198-743X(20)30706-0/fulltext

In your revision ensure you cite all your sources (including your own works), and quote or rephrase any duplicated text outside the methods section. Further consideration is dependent on these concerns being addressed :Done. 

Reviewer 1

*Do you have anti- SARS-CoV-2 IgM antibodies especially in PCR positive samples? In SARS-CoV-2 infections, IgM and IgG antibodies can arise nearly simultaneously in serum within 2 to 3 weeks after illness onset. Thus, detection of IgM without IgG is uncommon (CDC, 2020). That is why we used the ROCHE chemiluminescence immunoassay (Elecsys anti-SARS-COV2 total) which is qualitative assay that is used to detect the total antibodies (including IgG) against SARS-COV2. 

*The discrepancy between the PCR positive cases and antibodies negative needs to be addressed and explained since about half of the PCR positive patients are antibodies negative. Is it because of early testing and if so what about the anti- SARS-CoV-2 IgM antibodies, or because of the false positive PCR or the false negative antibodies:

 The discrepancy between the PCR positive cases and antibodies negative may be explained by:

1) Antibodies usually start to appear 7 to 10 days post-infection and even become detectable during the third week as recommended by the CDC. By that time the patient infectiousness start to decline. The antibodies determine whether the patient has been previously exposed to infection but cannot be used to assess the current infection status.

2) `33% of COVID 19 patients were found to have no detectable antibodies. That is why the CDC recommended not to use the antibody test neither in the diagnosis of COVID-19 cases nor in the determination of immunity.

Refer to CDC Interim Guidelines for COVID-19 Antibody Testing

*Do you have samples to follow some of the PCR positive antibodies negative to evaluate if they will be seroconverted : This was beyond the scope of the study.

*Cross-reactive antibodies to other Corona viruses should be considered especially for PCR negative antibodies positive samples and may be needed to be discussed or confirmed with other serological kits The analytical specificity of the Elecsys anti-SARS COV2 assay is 99.5% as determined by the manufacturer (Elecsys anti-SARS COV2 assay package insert). A total of 792 potentially cross-reacting samples were tested and no-cross reactivity has been reported with the common cold panel (number of tested samples = 40) nor the coronavirus panel (number of tested samples = 40).

Reviewer 2

 *Introduction COV-2 over the entire population is likely much higher, and accordingly the mortality rate should be fundamentally lower.

What are these numbers? Agencies sited? 

The total number of screening tests done in Egypt has been substantially lower than that done in other countries 

*The number at writing the manuscript should be included 

Numbers of daily PCR test that conducted daily at the time of writing the manuscript was added " Page 2 line 14"

as duration antibodies in patients after infection

*this statement is incomplete The statement was corrected 

Gynecology and Obstetrics, Oncology, Pediatrics, Psychiatry, Internal Medicine, surgical, cardiothoracic and Geriatrics

Are these different hospitals or departments within the same hospital Yes, these are different hospitals.

 Ain Shams university hospitals include 7 hospitals (surgery hospital, internal medicine hospital, pediatrics hospital, obstetrics, and gynecology hospital, emergency hospital , and geriatrics hospital), and multiple specific units and centers as clinical toxicology center, psychiatric center, bone marrow transplant unit, digestive endoscopy unit and clinical research unit. It involves more than 3000 inpatient beds, in addition to ICUs at the different hospitals.

Methodology:

*Nucleic acid extraction: Viral RNA was extracted using the Viasure RNA/DNA extraction kit (CerTest Biotec, Spain) as follows: � Each sample was mixed well by gentle vortexing and a volume of 300ul was transferred into a deep-well plate. � A total volume of 314ul lysing solution containing 300ul of lysis buffer, 10ul proteinase K solution and 4ul of carrier RNA was added into each sample well. � Sample plate was then loaded onto the Chemagic 360 automatic extractor (Perkin Elmer, Germany). Viral RNA was then automatically extracted by binding to the surface of magnetic beads. Impurities such as salts, metabolites, and soluble macromolecular cellular components were efficiently removed by a series of quick washing steps. � Unless immediately processed, extracted RNA was kept at -20�C.

Comment : follow manufacturers protocol Done 

Ethical considerations: 

The protocol of the research was approved by the University Ethical Research Committee

Comment: Insert the approval number Done 

Results: The current research enrolled 4313 subjects during the study period. A total of 4008 and 2951 patients had the PCR test and the antibody assay done, respectively. Females constituted 56% of the study sample. Adults and middle age represented around 60% of the sample. The vast majority of patients (91.3%) did not complain of any related COVID-19 symptoms.

*Comment : table or figure should be stated: Done.

Males showed higher risk for getting the COVID-19 infection as detected by positive PCR (OR adjusted for age was 1.45, 95% CI 1.06 8-1.98). On analyzing the adult group separately for comorbid conditions, males preserved their risk differential after adjustment for comorbid conditions (Diabetes and hypertension). Age and comorbid conditions did not show any significant relation to PCR positivity rate

*Comments: no tables or figures :Done.

Attachment Submitted filename: Response to reviwers.docx Click here for additional data file.

# Peer review: Decision Letter 1

**DOI:** 10.1371/journal.pone.0254581.r003

**Authors:** Jason T. Blackard

30 Jun 2021

SARS-CoV-2 PCR positivity rate and seroprevalence of related antibodies among a sample of patients in Cairo: Pre-wave 2 results of a screening program in a university hospital

PONE-D-21-06534R1

Dear Dr. Mossad,

We’re pleased to inform you that your manuscript has been judged scientifically suitable for publication and will be formally accepted for publication once it meets all outstanding technical requirements.

Within one week, you’ll receive an e-mail detailing the required amendments. When these have been addressed, you’ll receive a formal acceptance letter and your manuscript will be scheduled for publication.

An invoice for payment will follow shortly after the formal acceptance. To ensure an efficient process, please log into Editorial Manager at http://www.editorialmanager.com/pone/, click the 'Update My Information' link at the top of the page, and double check that your user information is up-to-date. If you have any billing related questions, please contact our Author Billing department directly at authorbilling@plos.org.

If your institution or institutions have a press office, please notify them about your upcoming paper to help maximize its impact. If they’ll be preparing press materials, please inform our press team as soon as possible -- no later than 48 hours after receiving the formal acceptance. Your manuscript will remain under strict press embargo until 2 pm Eastern Time on the date of publication. For more information, please contact onepress@plos.org.

Kind regards,

Jason T. Blackard, PhD

Academic Editor

PLOS ONE

Additional Editor Comments (optional):

None

Reviewers' comments:

Reviewer's Responses to Questions

**Comments to the Author**

1. If the authors have adequately addressed your comments raised in a previous round of review and you feel that this manuscript is now acceptable for publication, you may indicate that here to bypass the “Comments to the Author” section, enter your conflict of interest statement in the “Confidential to Editor” section, and submit your "Accept" recommendation.

Reviewer #1: All comments have been addressed

Reviewer #2: All comments have been addressed

**********

2. Is the manuscript technically sound, and do the data support the conclusions?

The manuscript must describe a technically sound piece of scientific research with data that supports the conclusions. Experiments must have been conducted rigorously, with appropriate controls, replication, and sample sizes. The conclusions must be drawn appropriately based on the data presented. 

Reviewer #1: Yes

Reviewer #2: Yes

**********

3. Has the statistical analysis been performed appropriately and rigorously? 

Reviewer #1: Yes

Reviewer #2: I Don't Know

**********

4. Have the authors made all data underlying the findings in their manuscript fully available?

The PLOS Data policy requires authors to make all data underlying the findings described in their manuscript fully available without restriction, with rare exception (please refer to the Data Availability Statement in the manuscript PDF file). The data should be provided as part of the manuscript or its supporting information, or deposited to a public repository. For example, in addition to summary statistics, the data points behind means, medians and variance measures should be available. If there are restrictions on publicly sharing data—e.g. participant privacy or use of data from a third party—those must be specified.

Reviewer #1: Yes

Reviewer #2: Yes

**********

5. Is the manuscript presented in an intelligible fashion and written in standard English?

PLOS ONE does not copyedit accepted manuscripts, so the language in submitted articles must be clear, correct, and unambiguous. Any typographical or grammatical errors should be corrected at revision, so please note any specific errors here.

Reviewer #1: Yes

Reviewer #2: Yes

**********

6. Review Comments to the Author

Please use the space provided to explain your answers to the questions above. You may also include additional comments for the author, including concerns about dual publication, research ethics, or publication ethics. (Please upload your review as an attachment if it exceeds 20,000 characters)

Reviewer #1: The authors responded adequately to the comments raised by the reviewers. They addressed the main concerns

Reviewer #2: (No Response)

**********

7. PLOS authors have the option to publish the peer review history of their article (what does this mean?). If published, this will include your full peer review and any attached files.

If you choose “no”, your identity will remain anonymous but your review may still be made public.

**Do you want your identity to be public for this peer review?** For information about this choice, including consent withdrawal, please see our Privacy Policy.

Reviewer #1: **Yes: **Mohamed Tarek M Shata

Reviewer #2: No

# Editor report: Acceptance letter

**DOI:** 10.1371/journal.pone.0254581.r004

**Authors:** Jason T. Blackard

5 Jul 2021

PONE-D-21-06534R1 

SARS-CoV-2 PCR positivity rate and seroprevalence of related antibodies among a sample of patients in Cairo: Pre-wave 2 results of a screening program in a university hospital 

Dear Dr. Mossad:

I'm pleased to inform you that your manuscript has been deemed suitable for publication in PLOS ONE. Congratulations! Your manuscript is now with our production department. 

If your institution or institutions have a press office, please let them know about your upcoming paper now to help maximize its impact. If they'll be preparing press materials, please inform our press team within the next 48 hours. Your manuscript will remain under strict press embargo until 2 pm Eastern Time on the date of publication. For more information please contact onepress@plos.org.

If we can help with anything else, please email us at plosone@plos.org. 

Thank you for submitting your work to PLOS ONE and supporting open access. 

Kind regards, 

PLOS ONE Editorial Office Staff

on behalf of

Dr. Jason T. Blackard 

Academic Editor

PLOS ONE
